# Glycemic Dysregulations Are Associated With Worsening Cognitive Function in Older Participants at High Risk of Cardiovascular Disease: Two-Year Follow-up in the PREDIMED-Plus Study

**DOI:** 10.3389/fendo.2021.754347

**Published:** 2021-10-29

**Authors:** Carlos Gómez-Martínez, Nancy Babio, Jordi Júlvez, Nerea Becerra-Tomás, Miguel Á. Martínez-González, Dolores Corella, Olga Castañer, Dora Romaguera, Jesús Vioque, Ángel M. Alonso-Gómez, Julia Wärnberg, José A. Martínez, Luís Serra-Majem, Ramón Estruch, Francisco J. Tinahones, José Lapetra, Xavier Pintó, Josep A. Tur, José López-Miranda, Aurora Bueno-Cavanillas, José J. Gaforio, Pilar Matía-Martín, Lidia Daimiel, Vicente Martín-Sánchez, Josep Vidal, Clotilde Vázquez, Emilio Ros, Søren Dalsgaard, Carmen Sayón-Orea, José V. Sorlí, Rafael de la Torre, Itziar Abete, Lucas Tojal-Sierra, Francisco J. Barón-López, Noelia Fernández-Brufal, Jadwiga Konieczna, Antonio García-Ríos, Emilio Sacanella, M. Rosa Bernal-López, José M. Santos-Lozano, Cristina Razquin, Andrea Alvarez-Sala, Albert Goday, M. Angeles Zulet, Jessica Vaquero-Luna, Javier Diez-Espino, Aida Cuenca-Royo, Fernando Fernández-Aranda, Mònica Bulló, Jordi Salas-Salvadó

**Affiliations:** ^1^Universitat Rovira i Virgili, Departament de Bioquímica i Biotecnologia, Unitat de Nutrició Humana, Reus, Spain; ^2^Institut d’Investigació Sanitària Pere Virgili (IISPV), Hospital Universitari San Joan de Reus, Reus, Spain; ^3^Consorcio CIBER, M.P. Fisiopatología de la Obesidad y Nutrición (CIBERObn), Instituto de Salud Carlos III (ISCIII), Madrid, Spain; ^4^Nutrition Unit, University Hospital of Sant Joan de Reus, Reus, Spain; ^5^Department of Preventive Medicine and Public Health, Instituto de Investigación Sanitaria de Navarra (IdISNA), University of Navarra, Pamplona, Spain; ^6^Department of Nutrition, Harvard T.H. Chan School of Public Health, Boston, MA, United States; ^7^Department of Preventive Medicine, University of Valencia, Valencia, Spain; ^8^Cardiovascular Risk and Nutrition Research Group (CARIN), Hospital del Mar Research Institute (IMIM), Barcelona, Spain; ^9^Health Research Institute of the Balearic Islands (IdISBa), University Hospital Son Espases, Palma, Spain; ^10^CIBER de Epidemiología y Salud Pública (CIBERESP), ISCIII, Madrid, Spain; ^11^Nutritional Epidemiology Unit, Miguel Hernandez University, Alicante, Spain; ^12^Instituto de Investigación Sanitaria y Biomédica de Alicante-Universidad Miguel Hernández (ISABIAL-UMH), Alicante, Spain; ^13^Bioaraba Health Research Institute, Osakidetza Basque Health Service, Araba University Hospital, University of the Basque Country Universidad del País Vasco / Euskal Herriko Unibertsitatea (UPV/EHU), Vitoria-Gasteiz, Spain; ^14^EpiPHAAN Research Group, School of Health Sciences, Instituto de Investigación Biomédica de Málaga (IBIMA), University of Malaga, Malaga, Spain; ^15^Department of Nutrition, Food Science and Physiology, Instituto de Investigación Sanitaria de Navarra (IdISNA), University of Navarra, Pamplona, Spain; ^16^Cardiometabolic Nutrition Group, Precision Nutrition and Cardiometabolic Health Program, IMDEA Food, Campus de Excelencia Internacional Universidad Autónoma de Madrid + Consejo Superior de Investigaciones Científicas (CEI UAM + CSIC), Madrid, Spain; ^17^Research Institute of Biomedical and Health Sciences (IUIBS), Preventive Medicine Service, Centro Hospitalario Universitario Insular Materno Infantil (CHUIMI), Canarian Health Service, University of Las Palmas de Gran Canaria, Las Palmas, Spain; ^18^Department of Internal Medicine, Institut d’Investigacions Biomèdiques August Pi Sunyer (IDIBAPS), Hospital Clinic, University of Barcelona, Barcelona, Spain; ^19^Department of Endocrinology, Instituto de Investigación Biomédica de Málaga (IBIMA), Virgen de la Victoria Hospital, University of Malaga, Malaga, Spain; ^20^Research Unit, Department of Family Medicine, Distrito Sanitario Atención Primaria Sevilla, Sevilla, Spain; ^21^Lipids and Vascular Risk Unit, Internal Medicine, Hospital Universitario de Bellvitge-IBIDELL, Hospitalet de Llobregat, Barcelona, Spain; ^22^Universitat de Barcelona, Barcelona, Spain; ^23^Research Group on Community Nutrition & Oxidative Stress, University of Balearic Islands-Instituto Universitario de Investigación en Ciencias de la Salud (IUNICS) & Health Research Institute of the Balearic Islands (IdISBa), Palma de Mallorca, Spain; ^24^Department of Internal Medicine, Maimonides Biomedical Research Institute of Cordoba (IMIBIC), Reina Sofia University Hospital, University of Cordoba, Cordoba, Spain; ^25^Department of Preventive Medicine, University of Granada, Granada, Spain; ^26^Instituto de Investigación Biosanitaria ibs.GRANADA, Granada, Spain; ^27^Departamento de Ciencias de la Salud, Instituto Universitario de Investigación en Olivar y Aceites de Oliva, Universidad de Jaén, Jaén, Spain; ^28^Department of Endocrinology and Nutrition, Instituto de Investigación Sanitaria Hospital Clínico San Carlos (IdISSC), Madrid, Spain; ^29^Nutritional Control of the Epigenome Group, Precision Nutrition and Obesity Program, IMDEA Food, CEI UAM + CSIC, Madrid, Spain; ^30^Institute of Biomedicine (IBIOMED), University of León, León, Spain; ^31^CIBER Diabetes y Enfermedades Metabólicas (CIBERDEM), ISCIII, Madrid, Spain; ^32^Departament of Endocrinology, Instituto de Investigaciones Biomédicas August Pi i Sunyer (IDIBAPS), Hospital Clínic, University of Barcelona, Barcelona, Spain; ^33^Department of Endocrinology, Fundación Jiménez-Díaz, Madrid, Spain; ^34^Lipid Clinic, Department of Endocrinology and Nutrition, IDIBAPS, Hospital Clínic, Barcelona, Spain; ^35^Lundbeck Foundation Initiative for Integrative Psychiatric Research, iPSYCH, Aarhus, Denmark; ^36^National Centre for Register-Based Research, Aarhus University, Aarhus, Denmark; ^37^Integrated Pharmacology and Systems Neurosciences Research Group, Hospital del Mar Medical Research Institute (IMIM), Barcelona, Spain; ^38^Departamento de Ciencias Experimentales y de la Salud (CEXS), Universitat Pompeu Fabra, Barcelona, Spain; ^39^Centro de Salud San Fermín, Elche, Spain; ^40^Department of Internal Medicine, Regional University Hospital of Malaga, Instituto de Investigación Biomédica de Málaga (IBIMA), University of Malaga, Malaga, Spain; ^41^Gerencia de Atención Primaria Servicio Navarro de Salud-Osasunbidea, Navarra, Spain; ^42^Department of Psychiatry, University Hospital of Bellvitge-Instituto de Investigación Biomédica de Bellvitge (IDIBELL), Barcelona, Spain; ^43^Department of Clinical Sciences, School of Medicine and Health Sciences, University of Barcelona, Barcelona, Spain

**Keywords:** cognitive function, diabetes duration, glycated (glycosylated) hemoglobin, insulin resistance, type 2 diabetes, prediabetes

## Abstract

**Introduction:**

Type 2 diabetes has been linked to greater cognitive decline, but other glycemic parameters such as prediabetes, diabetes control and treatment, and HOMA-IR and HbA_1c_ diabetes-related biomarkers have shown inconsistent results. Furthermore, there is limited research assessing these relationships in short-term studies. Thus, we aimed to examine 2-year associations between baseline diabetes/glycemic status and changes in cognitive function in older participants at high risk of cardiovascular disease.

**Methods:**

We conducted a 2-year prospective cohort study (n=6,874) within the framework of the PREDIMED-Plus study. The participants (with overweight/obesity and metabolic syndrome; mean age 64.9 years; 48.5% women) completed a battery of 8 cognitive tests, and a global cognitive function Z-score (GCF) was estimated. At baseline, participants were categorized by diabetes status (no-diabetes, prediabetes, and <5 or ≥5-year diabetes duration), and also by diabetes control. Furthermore, insulin resistance (HOMA-IR) and glycated hemoglobin (HbA_1c_) levels were measured, and antidiabetic medications were recorded. Linear and logistic regression models, adjusted by potential confounders, were fitted to assess associations between glycemic status and changes in cognitive function.

**Results:**

Prediabetes status was unrelated to cognitive decline. However, compared to participants without diabetes, those with ≥5-year diabetes duration had greater reductions in GCF (β=-0.11 (95%CI -0.16;-0.06)], as well as in processing speed and executive function measurements. Inverse associations were observed between baseline HOMA-IR and changes in GCF [β=-0.0094 (95%CI -0.0164;-0.0023)], but also between HbA_1c_ levels and changes in GCF [β=-0.0085 (95%CI -0.0115, -0.0055)], the Mini-Mental State Examination, and other executive function tests. Poor diabetes control was inversely associated with phonologic fluency. The use of insulin treatment was inversely related to cognitive function as measured by the GCF [β=-0.31 (95%CI -0.44, -0.18)], and other cognitive tests.

**Conclusions:**

Insulin resistance, diabetes status, longer diabetes duration, poor glycemic control, and insulin treatment were associated with worsening cognitive function changes in the short term in a population at high cardiovascular risk.

**Clinical Trial Registration:**

http://www.isrctn.com/ISRCTN89898870, identifier ISRCTN: 89898870.

## Introduction

Type 2 diabetes is an important public health problem worldwide. In 2019, the International Diabetes Federation estimated that ∼463 million people were living with diabetes (and 374 million had prediabetes), of whom one-third were >65 years old, and this figure is expected to rise to 700 million by 2045 (1). Diabetes mellitus is not only among the top 10 causes of death worldwide (2), but is also a risk factor for blindness, renal failure, and lower limb amputation, overall decreasing quality of life (2). As well, over 50 million people worldwide live with dementia, a form of cognitive impairment, and this number is expected to triple by 2050 (3). Cognitive impairment, characterized by loss of memory, concentration and reduced ability to learn new things, affecting everyday life, is relatively common and is a costly condition for the health system (3).

Meta-analyses and longitudinal studies of population-based cohorts have shown an increased risk of cognitive dysfunction in people with metabolic syndrome, prediabetes and diabetes (4–6). Specifically, type 2 diabetes has been related to deficits in different cognitive domains (7) and to accelerated cognitive decline, especially in psychomotor speed, memory and executive functions (8). However, some prospective studies have failed to confirm these associations (9, 10). Also, the relationship between cognitive decline and metabolic syndrome, prediabetes, insulin resistance and glycemic control is less well understood (4, 6, 11). Therefore, more studies are warranted to determine if glycemic dysregulations before diabetes onset may affect cognition in order to establish early strategies of prevention-focused on these populations.

Risk factors for cognitive decline when type 2 diabetes has been already established are also of great interest because consideration of these could help screen individuals with diabetes who may particularly benefit from intensive and suitable treatment strategies. The risk of accelerated cognitive decline in type 2 diabetes has been reported by some studies to be dependent on both disease duration and glycemic control (5, 12). Glucose-lowering treatments have also been related to cognitive function in a few epidemiologic studies with moderate-quality evidence (6, 13). Therefore, more studies are required to increase the strength of the evidence for these associations.

Furthermore, there is a gap in the research relating to shorter follow-up studies assessing the aforementioned relationships. Majority of the research to date has been conducted with medium to long-term duration (from 4 to more years of follow-up) (5, 9). The PREDIMED-Plus study offers an unprecedented opportunity to evaluate cognitive changes, using a battery of cognitive tests, and several measurements of glycemic status in a large population at high cardiovascular disease risk in the shorter term (2 years).

The objectives of the present study were to examine longitudinal associations between glycemic status (diabetes status, control/treatment, and related biomarkers) and cognitive decline and impairment. We hypothesized that glycemic dysregulations would be negatively associated with changes in cognitive function.

## Materials and Methods

The present study is based on an observational prospective cohort design conducted within the framework of the PREDIMED-Plus study using 2 years of follow-up data. The PREDIMED-Plus study is a multicenter, randomized, parallel-group clinical trial conducted in Spain for primary cardiovascular disease prevention. Participants were randomized to an intensive weight loss intervention program based on an energy-restricted traditional Mediterranean diet, physical activity promotion and behavioral support (intervention group) or usual care consisting of general recommendations to follow an energy-unrestricted Mediterranean diet (control group). The study protocol has been described extensively elsewhere (14) and can be found at http://www.predimedplus.com. The trial was registered in 2014 at the International Standard Randomized Controlled Trial (http://www.isrctn.com/ISRCTN89898870).

### Study Population

Eligible participants were community-dwelling adults (55–75 years) with overweight/obesity (27≤ BMI <40 kg/m^2^) who met at least three criteria of metabolic syndrome (15). Exclusion criteria are reported elsewhere (14).

Participant recruitment was conducted between October 2013 and December 2016 in 23 Spanish health centers. A total of 6,874 candidates met eligibility criteria and were randomly allocated in a 1:1 ratio to the intervention or control groups, using a centrally controlled, computer-generated random-number internet-based system with stratification by center, sex, and age. Couples sharing the same household were randomized together, using the couple as unit of randomization. The flow-chart of the studied PREDIMED-Plus population is shown in **Supplementary Figure 1**.

All participants provided written informed consent, and the study protocol and procedures were approved by all the ethical committees of all participating institutions.

### Diabetes Status and Glycemic Measurements

At baseline fasting blood samples were collected and biochemical analyses were performed to determine fasting plasma glucose and glycated hemoglobin (HbA_1c_) by routine laboratory methods. Insulin was centrally measured by an electrochemiluminescence immunoassay using an Elecsys immunoanalyzer (Roche Diagnostics, Meylan, France). Insulin resistance was estimated at baseline using the Homeostasis Model Assessment of Insulin Resistance (HOMA-IR) index (16).

Prediabetes and diabetes were defined following the American Diabetes Association criteria (17). Diabetes was defined as a previous diagnosis of diabetes, HbA_1c_ ≥48 mmol/mol (6.5%), use of antidiabetic medication, or having fasting plasma glucose >126 mg/dl in both the screening and baseline visits. Self-reported diabetes duration was categorized in <5-year and ≥5-year diabetes duration. Prediabetes status was defined as HbA_1c_ being between 39 mmol/mol (5.7%) and 46 mmol/mol (6.4%), or having fasting plasma glucose between ≥100 mg/dl and ≤125 mg/dl. Participants who did not meet any of these parameters were categorized into the no-diabetes category. Furthermore, we categorized diabetes status in participants with diabetes (participants with <5-year and ≥5-year diabetes duration) and no-diabetes (participants with prediabetes and no-diabetes).

Glycated hemoglobin was used to categorize participants into those having “good” or “poor” diabetic control [HbA_1c_ <57 mmol/mol or ≥57 mmol/mol (7.4%)], respectively (17). Diabetes treatment was assessed at baseline using self-reported data on insulin, sulfonylureas, metformin or dipeptidyl peptidase-4 inhibitors (IDPP-4) use.

### Covariates

Covariates were evaluated at baseline by trained staff in a face-to-face interview using self-reported general questionnaires on socio-demographics (sex, age, level of education, and civil status), lifestyle (alcohol intake, smoking habits, physical activity, and Mediterranean diet adherence), and disease history. Baseline anthropometric variables (weight and height) were determined to estimate body mass index (BMI). Adherence to an energy-reduced Mediterranean diet was assessed using a 17-point diet score, adapted from a previously validated one (18). Leisure-time physical activity was estimated using a validated short version of the Minnesota Leisure-Time Physical Activity Questionnaire (19, 20). The depressive status risk was evaluated using the Beck Depression Inventory-II (21).

### Neuropsychological Assessment

A battery of 8 cognitive tests was administered at baseline and 2 years of follow-up by trained staff. The tests performed, Mini-Mental State Examination (MMSE), Clock Drawing Test (CDT), Digit Span Test forward (DST-f) and backward (DST-b) section, Verbal Fluency Test animals (VFT-a) and “p” (VFT-p) version, and Trail Making Test part A (TMT-A) and B (TMT-B) are described in **Supplementary Material 1**.

### Statistical Analyses

We used the December 2020 PREDIMED-Plus database. Descriptive variables are reported as means and standard deviation (SD) for continuous variables or numbers and percentages (%) for qualitative variables. Differences between diabetes status and baseline characteristics were examined using chi-square and one-way ANOVA, for qualitative and quantitative variables, respectively.

For longitudinal analysis, linear and logistic regression models were used, including only participants with complete cognitive data at baseline and 2 years of follow-up for each cognitive test analyzed. To facilitate comparisons across cognitive tests, Z-scores were generated for each cognitive score at baseline and after 2 years using the mean and SD of baseline data, as previously reported (5, 12). A global cognitive function Z-score (GCF) was obtained averaging all cognitive Z-scores at each time point, standardizing by the mean and SD of cognitive Z-scores at baseline.

Using linear regression analyses we examined the associations between baseline status and 2-year changes in cognitive Z-scores in relation to: a) HOMA-IR levels; b) diabetes status, no diabetes being the reference group; c) HbA_1c_ levels; d) glycemic control measured by HbA_1c_ in participants with diabetes, good glycemic control being the reference group; e) diabetes treatment in participants with diabetes, no treatment being the reference group. Two models were fitted to adjust linear and logistic regression analyses. Model 1 was adjusted for baseline sex, age (years), intervention group, and center size (with <250; 250-300, 300-400; >400 randomized participants). Model 2 was additionally adjusted for baseline education level (primary school; high school; college), civil status (single, divorced or separated; married; widower), physical activity (MET min/week), smoking habits (smoker; former smoker; never smoker), alcohol intake (g/day), 17-point Mediterranean diet score, BMI (kg/m^2^), hypertension (yes/no), hypercholesterolemia (yes/no), and depression (yes/no). Furthermore, Model 3 was fitted exclusively for antidiabetic treatments to further adjust for baseline diabetes control (good/poor) and diabetes duration (<5-year diabetes duration/≥5-year diabetes duration).

Logistic regression analyses were used to estimate odds ratios (OR) and 95% confidence intervals (95% CI), examining the 2-year risk for cognitive impairment in participants with normal cognitive performance at baseline by diabetes status, with no diabetes being the reference group. Cognitive function cut-offs were defined by the dichotomization of neuropsychological assessments at the respective visits. Cognitive impairment was defined as GCF ≤10^th^ percentile, MMSE ≤24 punctuation, CDT ≤4 punctuation, and VFT-a, VFT-p, DST-d, DST-b ≤ respective mean - 1.5*SD and TMT-A, TMT-B ≥ respective mean + 1.5*SD (22–25).

Interaction analyses between glycemic status (diabetes status, HOMA-IR, HbA_1c_, and glycemic control and treatment) and sex, age, hypertension and BMI for the GCF were performed by comparing the model with and without the interaction product using the likelihood ratio test.

Participants with missing data on covariables (always <1% missing) were imputed as either the mean of the group or into the subcategory with the highest frequency (26).

All analyses were conducted with robust estimates of the variance to correct for intracluster correlation. The data were analyzed using the Stata-14 software program (StataCorp). Statistical significance was set using the Benjamini-Hochberg false discovery rate correction procedure (27) at a Q-value <0.05.

## Results

### Descriptive Results

**Table 1** shows the baseline characteristics of the study population (n=6,874) according to diabetes status. A total of 20.9% of participants were classified as having no-diabetes, 48.6% prediabetes, 14.8% with <5-year diabetes duration, and 15.6% with ≥5-year diabetes duration. The mean age of the total population was 64.9 ± 4.9 years and 48.5% were women. Participants with ≥5-year diabetes duration were older, had lower education level and alcohol consumption, greater adherence to the Mediterranean diet and higher HbA_1c_ levels. They were also more likely to have hypertension, hypercholesterolemia and depressive symptoms. Participants with <5-year diabetes duration had greater prevalence of obesity and higher HOMA-IR levels, and were less likely to be a woman. Participants without diabetes were more likely to have a higher education level. All cognitive assessments showed significant differences across diabetes status and participants with ≥5-year diabetes duration with lower scores.

**Table 1:** Baseline characteristics by diabetes status.

Characteristics	Diabetes status				P-value
	No-Diabetes (n=1440)	Prediabetes (n=3341)	<5y Diabetes (n=1020)	≥5y Diabetes (n=1073)	
Age (years)	64.5 ± 4.92	65.0 ± 4.91	64.7 ± 4.98	65.5 ± 4.81	<0.001
Sex (women)	706 (49.03)	1703 (50.97)	435 (42.65)	491 (45.76)	<0.001
Intervention group	730 (50.69)	1632 (48.85)	503 (49.31)	541 (50.42)	0.623
Education level					<0.001
Primary school or less	653 (45.35)	1627 (48.70)	489 (47.94)	593 (55.27)	
High school	417 (28.96)	976 (29.21)	302 (29.61)	291 (27.12)	
College	370 (25.69)	738 (22.09)	229 (22.45)	189 (17.61)	
Civil status					0.803
Single, divorced or separated	199 (13.82)	440 (13.17)	123 (12.06)	135 (12.58)	
Married	1097 (76.18)	2546 (76.20)	797 (78.14)	821 (76.51)	
Widower	144 (10.00)	355 (10.63)	100 (9.80)	117 (10.90)	
Physical activity (MET min/week)	2508 ± 2433	2493 ± 2264	2344 ± 2140	2420 ± 2378	0.236
Current smoker					0.195
Smoker	170 (11.81)	418 (12.51)	138 (13.53)	131 (12.21)	
Former smoker	602 (41.81)	1434 (42.92)	463 (45.39)	484 (45.11)	
Never smoker	668 (46.39)	1434 (44.57)	419 (41.08)	458 (42.68)	
Alcohol consumption (g/day)	11.0 ± 14.2	11.6 ± 15.9	11.7 ± 15.6	9.8 ± 14.6	0.004
17-point Mediterranean diet score	8.51 ± 2.71	8.37 ± 2.70	8.64 ± 2.60	8.72 ± 2.55	0.001
BMI (kg/m²)	32.2 ± 3.46	32.6 ± 3.41	32.9 ± 3.49	32.6 ± 3.52	<0.001
HOMA-IR	3.91 ± 2.61	5.08 ± 3.14	6.65 ± 4.19	6.30 ± 4.45	<0.001
HbA_1c_ (mmol/mol)	36.4 ± 4.7	40.5 ± 3.5	49.3 ± 10.2	54.7 ± 13.1	<0.001
HbA_1c_ (%)	5.48 ± 0.43	5.86 ± 0.32	6.66 ± 0.93	7.16 ± 1.20	<0.001
Hypertension	1192 (82.78)	2764 (82.73)	855 (83.82)	947 (88.26)	<0.001
Hypercholesterolemia	966 (67.08)	2281 (68.27)	755 (74.02)	811 (75.58)	<0.001
Depressive symptomatology	281 (19.51)	667 (19.96)	226 (22.16)	253 (23.58)	0.029
**Cognitive assessments**	**Diabetes status**				
	**No-Diabetes**	**Prediabetes**	**<5y Diabetes**	**≥5y Diabetes**	
MMSE (n=6654)	28.3 ± 1.85	28.3 ± 1.86	28.2 ± 1.95	28 ± 2.10	<0.001
CDT (n=6659)	5.95 ± 1.29	5.96 ± 1.21	6.02 ± 1.12	5.76 ± 1.34	<0.001
DST-f (n=5867)	8.95 ± 2.59	8.78 ± 2.39	8.87 ± 2.48	8.52 ± 2.48	<0.001
DST-b (n= 5864)	5.28 ± 2.36	5.11 ± 2.20	5.19 ± 2.19	4.93 ± 2.15	0.043
VFT-a (n=6816)	16.4 ± 5.00	16.1 ± 4.75	16.1 ± 4.84	15.2 ± 4.65	<0.001
VFT-p (n=6816)	12.6 ± 4.62	12.4 ± 4.53	12 ± 4.35	11.4 ± 4.39	<0.001
TMT-A (n=6802)§	50.9 ± 28.0	52.3 ± 27.5	52.7 ± 30.2	56.2 ± 30.2	<0.001
TMT-B (n=6783)§	121.6 ± 68.6	128.0 ± 70.2	130.1 ± 72.3	144.2 ± 79.6	<0.001

<5y diabetes, less than 5 years diabetes duration; ≥5y diabetes, more than 5 years diabetes duration; GCF, Global Cognitive Function; MMSE, Mini-Mental State Examination; CDT, Clock Drawing Test; DST-f, Digit Span Test forward section; DST-b, Digit Span Test backward section; VFT-a, Verbal Fluency Test animal category; VFT-p, Verbal Fluency Test letter “p”; TMT-A, Trail Making Test part A; TMT-B, Trail Making Test part B.

§ Inverse neuropsychological assessment score.

Data are n (%) or mean ± SD for categorical and quantitative variables, respectively.

Only the participants reported in each neuropsychological assessment are available.

Chi-square is used for categorical variables and One-way ANOVA for quantitative variables.

### Diabetes Status and Related Biomarkers

**Table 2** shows the associations between baseline diabetes status and 2-year changes in cognitive Z-scores. Compared to participants without diabetes, no significant differences in the associations between prediabetes and cognitive tests were observed. Compared to participants without diabetes, those with <5-year diabetes duration displayed larger decrements in cognitive Z-scores measured by the GCF, VFT-a, VFT-p and TMT-B tests in model 1, but these associations were attenuated in model 2. Compared to participants without diabetes, those with ≥5-year diabetes duration displayed larger reductions in all cognitive assessments in model 1, except in the case of the CDT test (**Table 2**). These associations remained significant for the GCF score, and the VFT-a, VFT-p, TMT-A and TMT-B tests in model 2. Similar results were found when comparing participants with diabetes and no-diabetes, finding a larger 2-year decrease with the presence of type 2 diabetes in the MMSE score (**Supplementary Table 1**).

**Table 2:** Association between baseline diabetes status and changes in cognitive Z-scores.

Z-scores	Diabetes status	Model 1		Model 2	
		β (95% CI)	P-value	β (95% CI)	P-value
GCF	No-Diabetes (n=1023)	Ref.		Ref.	
	Prediabetes (n=2429)	-0.04 (-0.10, 0.03)	0.277	-0.01 (-0.04, 0.03)	0.756
	<5y Diabetes (n=667)	-0.12 (-0.20, -0.03)	0.008*	-0.04 (-0.09, 0.01)	0.109
	≥5y Diabetes (n=684)	-0.27 (-0.36, -0.18)	<0.001*	-0.11 (-0.16, -0.06)	<0.001*
MMSE	No-Diabetes (n=1187)	Ref.		Ref.	
	Prediabetes (n=2786)	-0.01 (-0.07, 0.05)	0.749	0.01 (-0.05, 0.06)	0.865
	<5y Diabetes (n=847)	-0.08 (-0.16, 0.01)	0.054	-0.05 (-0.13, 0.03)	0.209
	≥5y Diabetes (n=865)	-0.11 (-0.19, -0.02)	0.011*	-0.06 (-0.14, 0.02)	0.134
CDT	No-Diabetes (n=1189)	Ref.		Ref.	
	Prediabetes (n=2788)	0.01 (-0.06, 0.07)	0.874	0.01 (-0.05, 0.07)	0.780
	<5y Diabetes (n=846)	-0.01 (-0.09, 0.08)	0.843	0.01 (-0.08, 0.09)	0.847
	≥5y Diabetes (n=866)	-0.09 (-0.18, -0.01)	0.031	-0.06 (-0.14, 0.03)	0.171
DST-f	No-Diabetes (n=1072)	Ref.		Ref.	
	Prediabetes (n=2526)	-0.03 (-0.10, 0.05)	0.474	-0.01 (-0.08, 0.06)	0.725
	<5y Diabetes (n=702)	-0.08 (-0.17, 0.01)	0.087	-0.06 (-0.15, 0.03)	0.198
	≥5y Diabetes (n=716)	-0.12 (-0.21, -0.03)	0.012*	-0.07 (-0.16, 0.02)	0.126
DST-b	No-Diabetes (n=1072)	Ref.		Ref.	
	Prediabetes (n=2525)	-0.04 (-0.11, 0.03)	0.293	-0.02 (-0.09, 0.04)	0.528
	<5y Diabetes (n=702)	-0.07 (-0.16, 0.02)	0.116	-0.04 (-0.13, 0.04)	0.349
	≥5y Diabetes (n=716)	-0.11 (-0.20, -0.02)	0.014*	-0.05 (-0.14, 0.04)	0.251
VFT-a	No-Diabetes (n=1226)	Ref.		Ref.	
	Prediabetes (n=2866)	-0.07 (-0.13, -0.01)	0.033	-0.05 (-0.11, 0.01)	0.101
	<5y Diabetes (n=870)	-0.14 (-0.22, -0.05)	0.001*	-0.10 (-0.17, -0.02)	0.018
	≥5y Diabetes (n=889)	-0.25 (-0.33, -0.16)	<0.001*	-0.18 (-0.26, -0.10)	<0.001*
VFT-p	No-Diabetes (n=1227)	Ref.		Ref.	
	Prediabetes (n=2865)	-0.05 (-0.12, 0.02)	0.149	-0.03 (-0.09, 0.03)	0.348
	<5y Diabetes (n=870)	-0.13 (-0.21, -0.04)	0.005*	-0.08 (-0.16, 0.01)	0.060
	≥5y Diabetes (n=889)	-0.23 (-0.32, -0.14)	<0.001*	-0.15 (-0.23, -0.07)	<0.001*
TMT-A§	No-Diabetes (n=1226)	Ref.		Ref.	
	Prediabetes (n=2862)	-0.02 (-0.08, 0.04)	0.512	-0.03 (-0.09, 0.03)	0.323
	<5y Diabetes (n=869)	0.08 (0.01, 0.16)	0.037	0.05 (-0.02, 0.13)	0.185
	≥5y Diabetes (n=886)	0.20 (0.11, 0.29)	<0.001*	0.15 (0.06, 0.23)	0.001*
TMT-B§	No-Diabetes (n=1221)	Ref.		Ref.	
	Prediabetes (n=2859)	0.01 (-0.05, 0.07)	0.690	0.01 (-0.06, 0.06)	0.994
	<5y Diabetes (n=866)	0.11 (0.03, 0.20)	0.006*	0.08 (0.01, 0.16)	0.039
	≥5y Diabetes (n=883)	0.24 (0.15, 0.32)	<0.001*	0.17 (0.09, 0.25)	<0.001*

<5y diabetes, less than 5 years diabetes duration; ≥5y diabetes, more than 5 years diabetes duration; GCF, Global Cognitive Function; MMSE, Mini-Mental State Examination; CDT, Clock Drawing Test; DST-f, Digit Span Test forward section; DST-b, Digit Span Test backward section; VFT-a, Verbal Fluency Test animal category; VFT-p, Verbal Fluency Test letter “p”; TMT-A, Trail Making Test part A; TMT-B, Trail Making Test part B.

§ Inverse neuropsychological assessment score.

Model 1: adjusted for sex, age (in years), intervention group, and center size (<250; 250-300, 300-400; ≥400).

Model 2: further adjusted for baseline education level (primary school; secondary school; college), civil status (single, divorced or separated; married; widower), physical activity (MET min/week), smoking habits (smoker; former smoker; never smoker), alcohol intake (g/day, adding the quadratic term), 17-point Mediterranean diet score, BMI (kg/m²), hypertension (yes/no), hypercholesterolemia (yes/no), and depressive symptomatology (yes/no).

Beta coefficients were estimated using linear regression models with robust standard errors to account for intracluster correlations.

*Significant association after Benjamini-Hochberg correction.

**Supplementary Table 2** shows the odds ratio (95% CI) for cognitive impairment incidence after 2 years of follow-up in participants with normal cognitive performance at baseline. Compared with participants without diabetes, those with diabetes had a borderline significant 34% (95% CI 0.96;1.87) higher risk of cognitive impairment when assessed by the GCF Z-score, and a non-significant 30% (95%CI 1.01;1.68) higher risk of impairment based on the VFT-a test after the false discovery rate correction. No significant associations were found between diabetes status and cognitive impairment incidence in the rest of the cognitive tests.

**Table 3** shows the association between baseline HOMA-IR (per one unit increment) and changes in cognitive Z-scores after 2 years of follow-up after excluding those participants with insulin treatment. Significant inverse associations between HOMA-IR and changes in cognitive Z-scores measured by GCF and the DST-f and DST-b tests were found (model 2). No significant associations between insulin resistance and changes in cognitive Z-scores were found for the MMSE, CDT, VFT-a, VFT-p, TMT-A and TMT-B tests. Furthermore, a sensitivity analysis was conducted excluding those participants with insulin or sulfonylurea treatment (n=596). Compared with the results of **Table 3**, no changes in the direction of β coefficients or significances after the Benjamini-Hochberg correction were shown.

**Table 3:** Association between baseline HOMA-IR levels (per one unit increment) and changes in cognitive Z-scores.

Z-scores	Model 1		Model 2	
	β (95% CI)	P-value	β (95% CI)	P-value
GCF (n=4377)	-0.0140 (-0.0217, -0.0061)	<0.001*	-0.0094 (-0.0164, -0.0023)	0.009*
MMSE (n=5180)	-0.0040 (-0.0120, 0.0039)	0.322	-0.0006 (-0.0087, 0.0075)	0.884
CDT (n=5183)	-0.0006 (-0.0075, 0.0064)	0.868	-0.0006 (-0.0077, 0.0065)	0.862
DST-f (n=4560)	-0.0116 (-0.0195, -0.0037)	0.004*	-0.0091 (-0.0170, -0.0013)	0.023
DST-b (n=4559)	-0.0106 (-0.0184, -0.0028)	0.007*	-0.0082 (-0.0157, -0.0006)	0.035
VFT-a (n=5319)	-0.0072 (-0.0144, 0.0001)	0.051	-0.0050 (-0.0121, 0.0020)	0.163
VFT-p (n=5319)	-0.0065 (-0.0146, 0.0015)	0.111	-0.0042 (-0.0115, 0.0030)	0.249
TMT-A (n=5311)§	0.0070 (-0.0008, 0.0147)	0.077	0.0040 (-0.0037, 0.0117)	0.306
TMT-B (n=5301)§	0.0087 (0.0014, 0.0159)	0.019	0.0060 (-0.0007, 0.0127)	0.079

GCF, Global Cognitive Function; MMSE, Mini-Mental State Examination; CDT, Clock Drawing Test; DST-f, Digit Span Test forward section; DST-b, Digit Span Test backward section; VFT-a, Verbal Fluency Test animal category; VFT-p, Verbal Fluency Test letter “p”; TMT-A, Trail Making Test part A; TMT-B, Trail Making Test part B.

§ Inverse neuropsychological assessment score.

Participants with insulin treatment were excluded (n=320) from the analysis.

Model 1: adjusted for sex, age (in years), intervention group, and center size (<250; 250-300, 300-400; ≥400).

Model 2: further adjusted for baseline education level (primary school; secondary school; college), civil status (single, divorced or separated; married; widower), physical activity (MET min/week), smoking habits (smoker; former smoker; never smoker), alcohol intake (g/day, adding the quadratic term), 17-point Mediterranean diet score, BMI (kg/m²), hypertension (yes/no), hypercholesterolemia (yes/no), and depressive symptomatology (yes/no).

Beta coefficients were estimated using linear regression models with robust standard errors to account for intracluster correlations

*Significant association after Benjamini-Hochberg correction.

**Table 4** presents the association between baseline HbA_1c_ levels (per one mmol/mol increment) and 2-year changes in cognitive Z-scores. An inverse association was observed between baseline HbA_1c_ levels and the GCF score, as well as the MMSE, VFT-a, VFT-p, TMT-A and TMT-B tests. No significant associations were found for the CDT, DST-f and DST-b tests.

**Table 4:** Association between baseline HbA_1c_ levels (per one mmol/mol increment) and cognitive Z-scores changes.

Z-scores	Model 1		Model 2	
	β (95% CI)	P-value	β (95% CI)	P-value
GCF (n=4406)	-0.0085 (-0.0115, -0.0055)	<0.001*	-0.0056 (-0.0081, -0.0030)	<0.001*
MMSE (n=5162)	-0.0043 (-0.0071, -0.0015)	0.002*	-0.0029 (-0.0055, -0.0002)	0.035*
CDT (n=5166)	-0.0017 (-0.0043, 0.0009)	0.210	-0.0007 (-0.0032, 0.0019)	0.615
DST-f (n=4601)	-0.0030 (-0.0061, 0.0001)	0.058	-0.0015 (-0.0045, 0.0015)	0.330
DST-b (n=4600)	-0.0042 (-0.0072, -0.0013)	0.005*	-0.0023 (-0.0051, 0.0005)	0.114
VFT-a (n=5316)	-0.0071 (-0.0099, -0.0043)	<0.001*	-0.0051 (-0.0078, -0.0024)	<0.001*
VFT-p (n=5316)	-0.0087 (-0.0118, -0.0056)	<0.001*	-0.0063 (-0.0091, -0.0035)	<0.001*
TMT-A (n=5307)§	0.0074 (0.0045, 0.0103)	<0.001*	0.0053 (0.0025, 0.0081)	<0.001*
TMT-B (n=5296)§	0.0072 (0.0043, 0.0100)	<0.001*	0.0045 (0.0019, 0.0072)	0.001*

GCF, Global Cognitive Function; MMSE, Mini-Mental State Examination; CDT, Clock Drawing Test; DST-f, Digit Span Test forward section; DST-b, Digit Span Test backward section; VFT-a, Verbal Fluency Test animal category; VFT-p, Verbal Fluency Test letter “p”; TMT-A, Trail Making Test part A; TMT-B, Trail Making Test part B.

§ Inverse neuropsychological assessment score.

Missing data on HbA_1c_ (n=633).

Model 1: adjusted for sex, age (in years), intervention group, and center size (<250; 250-300, 300-400; ≥400).

Model 2: further adjusted for baseline education level (primary school; secondary school; college), civil status (single, divorced or separated; married; widower), physical activity (MET min/week), smoking habits (smoker; former smoker; never smoker), alcohol intake (g/day, adding the quadratic term), 17-point Mediterranean diet score, BMI (kg/m²), hypertension (yes/no), hypercholesterolemia (yes/no), and depressive symptomatology (yes/no).

Beta coefficients were estimated using linear regression models with robust standard errors to account for intracluster correlations.

*Significant association after Benjamini-Hochberg correction.

There were no significant interactions by sex, age, hypertension or BMI between the glycemic status (HOMA-IR, HbA_1c_ and glycemic control/treatment) and changes in the GCF score (all p>0.05). However, an interaction by age was found between diabetes status and changes in the GCF score (P=0.046). Compared to participants without diabetes, a larger decline in the GCF score was shown in those participants aged ≤65 years and presenting with prediabetes and <5-year and ≥5-year of diabetes duration, whereas participants aged >65 years with prediabetes showed increased performance in the GCF score. No associations were found between diabetes duration and the GCF score in participants aged >65 years.

### Diabetes Control and Treatment

**Supplementary Table 3** shows the association between baseline glycemic control (HbA_1c_ ≥57 mmol/mol or <57 mmol/mol) in participants with diabetes and 2-year changes in cognitive Z-scores. Compared to participants with good diabetes control, those with poor control showed a larger decrement in the VFT-p [β= -0.13 (95%CI -0.22;-0.04)] test (model 2). No associations between glycemic control and the rest of the cognitive tests were observed.

**Supplementary Table 4** shows the association between baseline insulin treatment in participants with diabetes and changes in cognitive Z-scores. Compared to participants without insulin treatment, those with insulin treatment showed a significantly greater decrease in cognitive function measured by the GCF score and the DST-f, DST-b, VFT-a, VFT-p, TMT-A and TMT-B tests. No associations were observed for the remaining cognitive tests assessed (MMSE and CDT). Concerning oral glucose medication use, sulfonylurea treatment was not significantly associated with an increase in the TMT-A (β= 0.22 [95%CI 0.07;0.38]) Z-score after the Benjamini-Hockberg correction (**Supplementary Table 5**). No significant associations were shown between the use of metformin or IDDP-4 and changes in cognitive Z-scores (**Supplementary Tables 6**, **7**, respectively). When the associations between diabetes treatment and cognitive function were further adjusted by diabetes duration or glycemic control, the results remained similar (model 3).

No significant interactions by sex, age, hypertension, and BMI were observed between diabetes control or treatment and changes in the GCF score.

## Discussion

To the best of our knowledge, this is the first prospective study investigating associations between glycemic status (diabetes status/control/treatment, and HOMA-IR and HbA_1c_ biomarkers) and cognitive function in a large cohort of older adults at risk high cardiovascular disease in a short period (2-year). In this community-based population, compared to participants without diabetes, those with diabetes showed a larger decline in several cognitive performance measurements. Additionally, longer duration of diabetes was associated with greater decreases in the scores of tests measuring processing speed and executive functions. Furthermore, poor diabetes control, the use of insulin treatment, and increases in HOMA-IR and HbA_1c_ levels were inversely associated with cognitive functioning.

Our results concur with those of meta-analyses of prospective studies, suggesting larger risk of cognitive decline in type 2 diabetes (6–8). The mechanisms explaining these associations remain largely unknown. Several risk factors for cognitive dysfunction in diabetes have been reported, such as hypertension or depression, but each of them appear to have weak isolated effects (28, 29). In order to control for these potential confounding factors, we have adjusted our statistical models for several recognized confounders.

Our findings are similar to those reported in other studies, suggesting a greater risk of cognitive decline in participants with type 2 diabetes, especially in relation to executive functions (5, 8, 30). Similarly, we found inverse associations in participants with diabetes and all the executive function-related tests, except in the case of the DST-b test, which measures working memory. Concerning memory function, we also assessed immediate verbal memory using the DST-f test, which was borderline inversely associated with the presence of diabetes. These results concur with those reported in a recent meta-analysis in which immediate (measured by the DST-f) and working memory (measured by the DST-b) were not associated in type 2 diabetes, while the other memory and executive function abilities assessed were reduced (8). Regarding visuospatial function, discrepancies in longitudinal studies have been reported in individuals with type 2 diabetes (31, 32). However, a small effect size in this function was reported in a meta-analysis conducted in 2014 (30). In our study, a non-significant inverse association between diabetes and the CDT test was observed, and longer follow-up of our population may be needed to observe a significant decline in this cognitive function.

Our results also showed that, compared to participants without diabetes, those with diabetes had a borderline increased risk of developing cognitive impairment as measured by the GCF score, even when the period of follow-up was only 2 years. Meta-analyses including prospective studies have shown an incidence of cognitive impairment in participants with type 2 diabetes (6, 33). However, the assessment of short-time periods were not commonly reported in regard to the association between type 2 diabetes and cognitive function, and it may be the reason for the discrepancies observed between the aforementioned meta-analyses and our study.

As far as we know, no longitudinal studies have been conducted assessing associations between diabetes status and cognitive decline, while also considering both the prediabetes status and the duration of diabetes. Longitudinal cohort studies have shown contradictory results regarding the association of prediabetes with cognition (5, 12, 31, 34), which can be explained by the different range of ages and sample sizes, the tests and cognitive domains assessed, and the length of follow-up. Concerning diabetes duration, our results are in line with other longitudinal studies in which higher rates of cognitive decline were described in individuals with longer diabetes duration (5, 12).

The observed interaction of the GCF score with age in prediabetes has not been previously reported in the literature and cannot be explained by a specific mechanism. We cannot rule out that this interaction was a random finding and it is a result that requires further investigation.

Several mechanisms have been suggested to explain the association between diabetes status and control with changes in cognitive functioning. Among them, insulin resistance, hyperglycemic excursions and glycemic control have received much attention. Insulin resistance linked to low-grade inflammation is a factor contributing to the onset of diabetes, that appears to play a key role in the cognitive impairment associated with obesity and diabetes, given the role that insulin has in the brain promoting neuronal survival and synaptic plasticity and inhibiting apoptosis and neuroinflammation (35). In the case of peripheral insulin resistance and type 2 diabetes, a decrease in insulin permeation through the blood-brain barrier was observed, leading to a smaller amount of insulin reaching the brain, thus impairing neuronal activation and inducing changes in synaptic plasticity, neuronal apoptosis and neuroinflammation, all responsible for cognitive deterioration (35).

Longitudinal studies linking insulin resistance, as measured by HOMA-IR, and cognitive decline have shown discrepancies. In an older U.S. population with 8 years of follow-up, baseline HOMA-IR was not associated with changes in global cognitive function (36). However, in surviving patients with coronary heart disease, baseline HOMA-IR was associated with subsequent poorer cognitive performance on the composite cognitive score over 15 years (37). Our results were in line with those of the latter study, as we also observed an inverse association between baseline HOMA-IR and changes in cognitive performance using a global cognitive function score.

Additional mechanisms explaining the deleterious association of diabetes on cognitive functioning include hyperglycemic status and glycemic excursions. Increased HbA_1c_ levels or high levels of repeated glucose measurements over time have been linked to cognitive decline and an increased risk of dementia in people without diabetes (38). In our study, no associations between HbA_1c_ levels and changes in cognitive function were observed in participants without diabetes (data not shown). Nevertheless, when HbA_1c_ was measured as a continuous variable, we found negative associations between high baseline values in HbA_1c_ levels and all the cognitive tests measured, except in the case of the CDT and the DSTs, thus aligning with findings from recent studies (34, 36).

When diabetes is established, increased HbA_1c_ levels have been linked to diabetes-associated cognitive decline and dementia, but the strength of these relationships is weak (11). In our study, compared to participants with good diabetes control, those with poor control showed a larger 2-year decrease in cognitive performance measured by the VFT-p test, but this association was not observed in the case of the GCF score and other cognitive assessments. Unlike other typical diabetic end-organ complications, no clear evidence exists that the increased risk of cognitive impairment can be attributed solely to hyperglycemic excursions and glycemic control (11). For example, the ACCORD MIND trial (39), which compared intensive with standard treatment with the aim to lower HbA_1c_ in people with long-standing type 2 diabetes, found no association between the intervention and cognitive function.

Several other mechanisms have been implicated in diabetes-related cognitive decline and dementia. For example, type 2 diabetes has substantial adverse effects on blood vessels and the heart (40), leading to an increased risk of stroke and small cerebral vessel disease. Indeed, neuropathological studies also report an increased burden of cerebrovascular lesions, especially of lacunar type, in people with diabetes (41).

Observational studies have reported that some glucose-lowering medications may have a potential beneficial or deleterious relationship with cognition (6, 13). In our study, contrary to other results showing improved cognitive function (13), no associations between metformin and cognition were observed, as well this was not observed for IDDP-4 or sulfonylureas use. However, in line with findings of recent meta-analyses, insulin-treated participants showed larger cognitive decline than those not treated with insulin (6, 13). This could be explained by the fact that these individuals tend to have worse glycemic control and larger risk of hypoglycemia, a condition that has been linked to cognitive decline and dementia risk (42, 43).

It is worth mentioning a strength of the present study is the novelty of being one of the largest population-based studies longitudinally and concurrently exploring relationships between glycemic status (diabetes status, markers of glucose metabolism, and diabetes control and treatment) and cognitive function in an older individuals at high cardiovascular risk. Moreover, this study suggests that larger follow-up periods are not required to observe associations between glycemic status and cognitive function. Nevertheless, the present findings should be considered in the context of some limitations. Firstly, although we adjusted the models for many potential confounding factors, there may be residual confounding factors not assessed, such as genetic susceptibility (APOE genotype). Unfortunately, genetic data was not available in all the PREDIMED-Plus population. Secondly, the PREDIMED-Plus study did not contemplate the use of neuroimaging, such as magnetic resonance imaging (MRI). Finally, our study has been conducted in older Mediterranean individuals with overweight/obesity at high risk of cardiovascular disease, and therefore we cannot extrapolate our results to other populations.

In conclusion, several glycemic dysregulations, such as insulin resistance measured by HOMA-IR, diabetes status, longer duration of diabetes, poor glycemic control and higher levels of HbA_1c_, and insulin treatment were associated with greater cognitive decline in older individuals with overweight/obesity at high cardiovascular disease risk in a short time period. We also reported that participants with type 2 diabetes had a borderline increased risk of developing cognitive impairment as measured by the GCF score, compared to those without diabetes. Therefore, it is clinically relevant to assess novel effective strategies at the initial stages of diabetes-related alterations in order to reduce the impact of cognitive dysfunction when these glycemic dysregulations are more pronounced.

## Data Availability Statement

There are restrictions on the availability of data for the PREDIMED-Plus trial, due to the signed consent agreements around data sharing, which only allow access to external researchers for studies following the project purposes. Requestors wishing to access the PREDIMED-Plus trial data used in this study can make a request to the PREDIMED-Plus trial Steering Committee chair: predimed_plus_scommitte@googlegroups.com. The request will then be passed to members of the PREDIMED-Plus Steering Committee for deliberation.

## Ethics Statement

The studies involving human participants were reviewed and approved by CEI Provincial de Málaga-Servicio Andaluz de Salud O01_feb_PR2 - Predimedplus nodo 1 CEI de los Hospitales Universitarios Virgen Macarena y Virgen del Rocío-Servicio Andaluz de Salud PI13/00673 CEIC Universidad de Navarra 053/2013 CEI de las Illes Balears - Conselleria de Salut Direcció General de Salut Publica i Consum IB 2242/14 PI CEIC del Hospital Clínic de Barcelona HCB/2016/0287 CEIC Parc de Salut Mar y IDIAP Jordi Gol PI13/120 CEIC del Hospital Universitari Sant Joan de Reus y IDIAB Jordi Gol 13-07-25/7proj2 CEI de la Provincia de Granada- Servicio Andaluz de Salud MAB/BGP/pg CEIC de la Fundacion Jiménez Díaz EC 26-14/IIS-FJD CEIC Universidad de Navarra 053/2013 CEIC Euskadi PI2014044 CEIC Corporativo de Atención Primaria de la Comunitat Valenciana 2011-005398-22 CEI Humana de la Universidad de las Palmas de Gran Canaria CEIH-2013-07 CEIC del Hospital de Bellvitge PR240/13 CEI de Cordoba-Junta de Salud 3078 CEI de la Fundación IMDEA Alimentación PI-012 CEIC Hospital Clínico San Carlos de Madrid-Piloto-CEIC Servicio Madrileño de salud-General 30/15 CEI Provincial de Málaga-Servicio Andaluz de Salud CEI de las Illes Balears - Conselleria de Salut Direcció General de Salut Publica i Consum IB 2251/14 PI CEIC del Hospital Clínic de Barcelona HCB/2017/0351 CEIC del Hospital General Universitario de Alicante CEIC PI2017/02 CEIC de la Investigación Biomédica de Andalucía (CCEIBA) CEI de la Universidad de León ÉTICA-ULE-014-2015. The participants provided their written informed consent to participate in this study.

## Author Contributions

The principal PREDIMED-Plus investigators (MM, JS-S, DC, JM, AA, JW, JVio, DR, JL-M, RE, FT, JL, LS-M, AB-C, JT, VM-S, XP, PM-M, JVid, CV, LD, and ER) contributed to study concept and design and to data extraction from the participants. CG, NBT, NB, JJ, and JS-S performed the statistical analyses. CG and JS-S drafted the manuscript. All authors reviewed the manuscript for important intellectual content and approved the final version to be published.

## Funding

This work was supported by the official Spanish Institutions for funding scientific biomedical research, CIBER Fisiopatología de la Obesidad y Nutrición (CIBEROBN) and Instituto de Salud Carlos III (ISCIII), through the Fondo de Investigación para la Salud (FIS), which is co-funded by the European Regional Development Fund (six coordinated FIS projects leaded by JS-S and JVid, including the following projects: PI13/00673, PI13/00492, PI13/00272, PI13/01123, PI13/00462, PI13/00233, PI13/02184, PI13/00728, PI13/01090, PI13/01056, PI14/01722, PI14/00636, PI14/00618, PI14/00696, PI14/01206, PI14/01919, PI14/00853, PI14/01374, PI14/00972, PI14/00728, PI14/01471, PI16/00473, PI16/00662, PI16/01873, PI16/01094, PI16/00501, PI16/00533, PI16/00381, PI16/00366, PI16/01522, PI16/01120, PI17/00764, PI17/01183, PI17/00855, PI17/01347, PI17/00525, PI17/01827, PI17/00532, PI17/00215, PI17/01441, PI17/00508, PI17/01732, PI17/00926, PI19/00957, PI19/00386, PI19/00309, PI19/01032, PI19/00576, PI19/00017, PI19/01226, PI19/00781, PI19/01560, PI19/01332, PI20/01802, PI20/00138, PI20/01532, PI20/00456, PI20/00339, PI20/00557, PI20/00886, PI20/01158); the Especial Action Project entitled: Implementación y evaluación de una intervención intensiva sobre la actividad física Cohorte PREDIMED-Plus grant to JS-S; the European Research Council (Advanced Research Grant 2014–2019; agreement #340918) granted to MM; the Recercaixa (agreement #2013ACUP00194) grant to JS-S; grants from the Consejería de Salud de la Junta de Andalucía (PI0458/2013, PS0358/2016, PI0137/2018); the PROMETEO/2017/017 grant from the Generalitat Valenciana; the SEMERGEN grant; The Horizon 2020 PRIME study (Prevention and Remediation of Insulin Multimorbidity in Europe; grant agreement #847879); JJ holds the Miguel Servet-II contract (CPII19/00015) awarded by the Instituto de Salud Carlos III (co-funded by the European Social Fund “Investing in your future”); JK was supported by the ‘FOLIUM’ programme within the FUTURMed project from the Fundación Instituto de Investigación Sanitaria Illes Balears (financed by 2017annual plan of the sustainable tourism tax and at 50% with charge to the ESF Operational Program 2014–2020 of the Balearic Islands); AÁ-S received a post-doctoral grant from the Generalitat Valenciana (APOSTD/2020/164); CG receives a predoctoral grant from the University of Rovira i Virgili (2020PMF-PIPF-37); We thank CERCA Programme/Generalitat de Catalunya for institutional support and partial support was also provided by SLT006/17/00246, funded by the Department of Health of the Generalitat de Catalunya by the calls “Acció instrumental de programes de recerca orientats en l’àmbit de la recerca i la innovació en salut” and “Pla estratègic de recerca i innovació en salut (PERIS)”; JS-S, senior author of this article, is partially supported by ICREA under the ICREA Academia program; None of the funding sources took part in the design, collection, analysis, interpretation of the data, or writing the report, or in the decision to submit the manuscript for publication.

## Conflict of Interest

JS-S serves on the board of the International Nut and Dried Fruit Council and receives grant support through this institution. He also served on the Executive Committee of the Instituto Danone, Spain, and on the Scientific Committee of the Danone International Institute. He has received research support from the Patrimonio Comunal Olivarero, Spain, and Borges S.A., Spain. He receives consulting fees or travel expenses from Eroski Foundation, the Instituto Danone, Spain, Mundipharma and Abbot Laboratories. ER reports grants, personal fees, non-financial support and others from California Walnut Commission and Alexion, personal fees, non-financial support and others from Ferrer International and Danone, and personal fees from Amarin, outside the submitted work. FF-A reports consultation fees from Novo Nordisk and editor-in-Chief honoraria from Wiley.

The remaining authors declare that the research was conducted in the absence of any commercial or financial relationships that could be construed as a potential conflict of interest.

## Publisher’s Note

All claims expressed in this article are solely those of the authors and do not necessarily represent those of their affiliated organizations, or those of the publisher, the editors and the reviewers. Any product that may be evaluated in this article, or claim that may be made by its manufacturer, is not guaranteed or endorsed by the publisher.

## Abbreviations

BMI, Body Mass Index; CDT, Clock Drawing Test; DST-b, Digit Span Test backward section; DST-f, Digit Span Test forward section; GCF, Global Cognitive Function; HbA_1c_, glycated hemoglobin; HOMA-IR, Homeostasis Model Assessment of Insulin Resistance; IDDP-4, dipeptidyl peptidase-4 inhibitors; MMSE, Mini-Mental State Examination; TMT-A, Trail Making Test A section; TMT-B, Trail Making Test B section; VFT-a, verbal Fluency Test animals category; VFT-p, Verbal Fluency Test letter p category.
