# Supplementary material for: Glycemic Dysregulations Are Associated With Worsening Cognitive Function in Older Participants at High Risk of Cardiovascular Disease: Two-Year Follow-up in the PREDIMED-Plus Study
Source: Front Endocrinol (Lausanne). 2021 Oct 29;12:754347. doi: 10.3389/fendo.2021.754347 (PMC8586462; doi:10.3389/fendo.2021.754347)
Supplement: Supplementary file 1 [file DataSheet_1.pdf]

Supplementary Material

SUPPLEMENTARY MATERIAL 1

The Mini-Mental State Examination (MMSE) validated for the Spanish population was used as a general cognitive screening test, which is an assessment of general cognitive function. The MMSE is divided into two sections, the first of which requires vocal responses only and covers orientation, memory, and attention, being the maximum score 21. The second part tests the ability to name, follow verbal and written commands, write a sentence spontaneously, and copy a complex polygon similar to a Bender-Gestalt figure, with a maximum possible score of 9. A high MMSE score indicates absence of cognitive decline [1].

The Clock Drawing Test (CDT) was used as a cognitive screening tool and the 7-point version, currently used for elderly Spanish populations, was performed [2]. Higher scores indicate better performance. The CDT assesses visuospatial, visuocontruction and memory capacities, as well as verbal and numerical knowledge [3].

The Digit Span Test (DST) of the WAIS-III Spanish version measuring attention and memory functions was used [4]. The Digit Span Test forward version (DST-f) requires participants to verbally repeat in the same order as provided, a number sequence, varying from 2 to 9 numbers, being representative of immediate memory. The DST backward version (DST-b) requires participants to verbally repeat in reverse order a number sequence, varying from 2 to 8 numbers, being representative of working memory function [5]. For both tests, the task is finished when the participant fails two attempts consecutively.

The Spanish Verbal Fluency Tests (VFTs) were used to assess language and executive function [6]. Specifically, we used the semantic VTF animal category version (VFT-a) and the phonemic VFT letter “p” version (VFT-p). Participants were instructed to say the maximum number of words related to the semantic field of animals for VFT-a and the maximum number of words starting with the letter “p” for VFT-p, having a time limit for both tests of 60 seconds. The total score was obtained from the row of properly stated words. The semantic VFT presents more influence on verbal abilities, whereas the phonologic VFT presents more influence on executive control ability [7].

The Trail Making Test (TMT) with normative data provided for the Spanish population was used [8]. The TMT consists of 25 circles spread over a sheet of paper and contains part A (TMT-A) and part B (TMT-B). In TMT-A, participants were asked to connect consecutive numbers (1–2–3–…) in the correct order by drawing a line. In TMT-B, they were asked to connect consecutive numbers and letters in an alternating numeric and alphabetic sequence (1–A–2–B–3–C–…). Each part is scored according to the time taken to complete the task, where more time spent indicates poorer performance. TMTs discriminate cognitive dysfunction [9] and TMT-A assesses attention and processing speed capacities [8] whereas TMT-B requires more executive function abilities such as cognitive flexibility [10].

REFERENCES

1. Folstein MF, Folstein SE, McHugh PR. “Mini-mental state”. A practical method for grading the cognitive state of patients for the clinician. J Psychiatr Res (1975) 12(3):189–98. doi:10.1016/0022-3956(75)90026-6

2. del Ser Quijano T, García de Yébenes MJ, Sánchez Sánchez F, Frades Payo B, Rodríguez Laso Á, Bartolomé Martínez MP, et al. Evaluación cognitiva del anciano. Datos normativos de una muestra poblacional española de más de 70 años. Med Clin (Barc) (2004) 122(19):727–40. doi:10.1157/13062190

3. Shulman KI. Clock-drawing: is it the ideal cognitive screening test? Int J Geriatr Psychiatry (2000) 15(6):548–61. doi:10.1002/1099-1166(200006)15:6<548::AID-GPS242>3.0.CO;2-U

4. TEA SA. WAIS-III: Escala de inteligencia de Wechsler para Adultos. Madrid: TEA (1999).

5. Wechsler D. Wechsler Memory Scale - Revised. Manual. San Antonio, TX: The Psychological Corporation (1987).

6. Peña-Casanova J, Quiñones-Úbeda S, Gramunt-Fombuena N, Quintana-Aparicio M, Aguilar M, Badenes D, et al. Spanish multicenter normative studies (NEURONORMA project): Norms for verbal fluency tests. Arch Clin Neuropsychol (2009) 24(4):395–411. doi: 10.1093/arclin/acp042

7. Shao Z, Janse E, Visser K, Meyer AS. What do verbal fluency tasks measure? Predictors of verbal fluency performance in older adults. Front Psychol (2014) 5(772). doi:10.3389/fpsyg.2014.00772

8. Llinàs-Reglà J, Vilalta-Franch J, López-Pousa S, Calvó-Perxas L, Torrents Rodas D, Garre-Olmo J. The Trail Making Test. Assessment (2017) 24(2):183–96. doi:10.1177/1073191115602552

9. Reitan RM. The relation of the Trail Making Test to organic brain damage. J Consult Psychol (1955) 19(5):393–4. doi:10.1037/h0044509

10. Kortte KB, Horner MD, Windham WK. The Trail Making Test, Part B: Cognitive Flexibility or Ability to Maintain Set? Appl Neuropsychol (2002) 9(2):106–9. doi:10.1207/S15324826AN0902_5

Supplementary Figure 1. Flow chart of participant selection for the present study population.

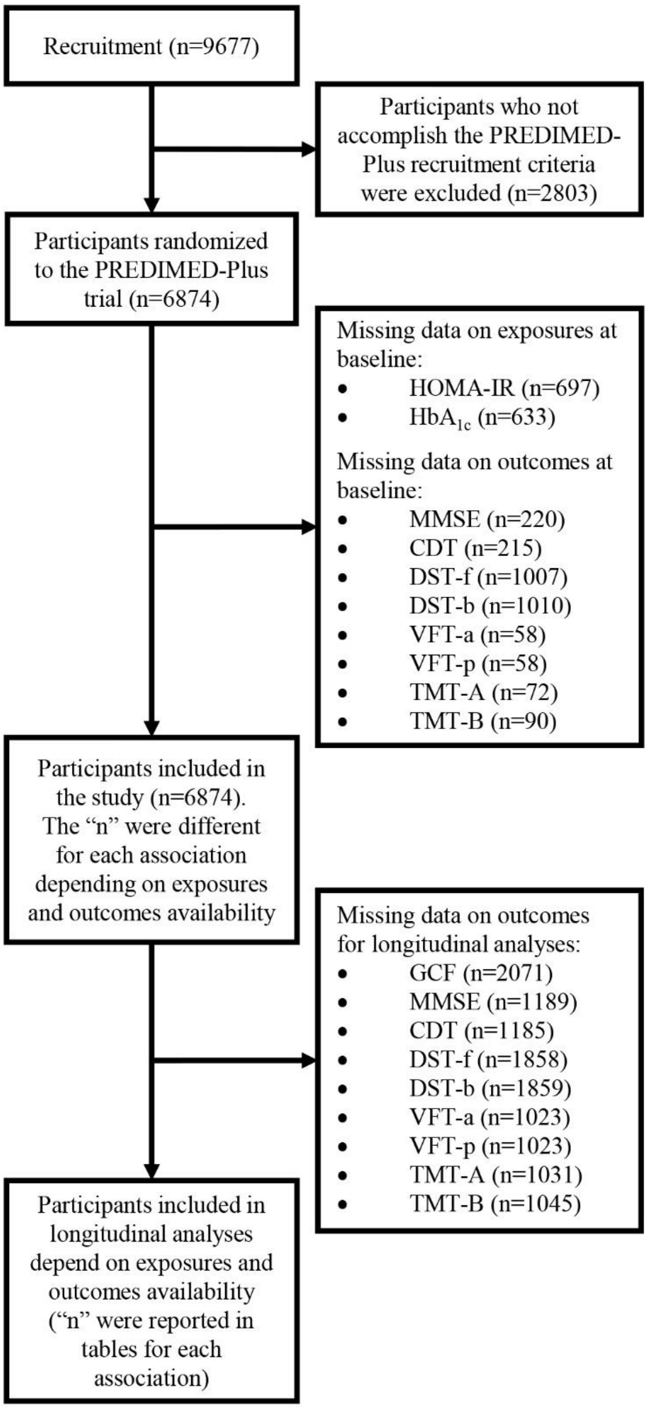

HOMA-IR, Homeostasis Model Assessment of Insulin Resistance; HbA1c, glycated hemoglobin; GCF, Global Cognitive Function; MMSE, Mini-Mental State Examination; CDT, Clock Drawing Test; DST-f, Digit Span Test forward section; DST-b, Digit Span Test backward section; VFT-a, Verbal Fluency Test animal category; VFT-p, Verbal Fluency Test letter "p"; TMT-A, Trail Making Test part A; TMT-B, Trail Making Test part B.

Supplementary Table 1 Association between dichotomized baseline diabetes status and changes in cognitive Z-scores.

Z-scores	Diabetes status	Model 1		Model 2	
		β (95% CI)	P-value	β (95% CI)	P-value
GCF	No-Diabetes (n=3452)	Ref.		Ref.	
	Diabetes (n=1351)	-0.17 (-0.23, -0.11)	<0.001*	-0.07 (-0.10, -0.04)	<0.001*
MMSE	No-Diabetes (n=3973)	Ref.		Ref.	
	Diabetes (n=1712)	-0.09 (-0.14, -0.03)	0.002*	-0.06 (-0.11, -0.01)	0.026*
CDT	No-Diabetes (n=3977)	Ref.		Ref.	
	Diabetes (n=1712)	-0.06 (-0.11, -0.01)	0.048	-0.03 (-0.09, 0.02)	0.234
DST-f	No-Diabetes (n=3598)	Ref.		Ref.	
	Diabetes (n=1418)	-0.08 (-0.14, -0.02)	0.007*	-0.05 (-0.11, 0.01)	0.058
DST-b	No-Diabetes (n=3597)	Ref.		Ref.	
	Diabetes (n=1418)	-0.07 (-0.12, -0.01)	0.022*	-0.03 (-0.09, 0.02)	0.265
VFT-a	No-Diabetes (n=3977)	Ref.		Ref.	
	Diabetes (n=1759)	-0.14 (-0.20, -0.09)	<0.001*	-0.10 (-0.15, -0.05)	<0.001*
VFT-p	No-Diabetes (n=4092)	Ref.		Ref.	
	Diabetes (n=1759)	-0.14 (-0.20, -0.09)	<0.001*	-0.09 (-0.15, -0.04)	<0.001*
TMT-A§	No-Diabetes (n=4088)	Ref.		Ref.	
	Diabetes (n=1755)	0.16 (0.10, 0.21)	<0.001*	0.12 (0.07, 0.17)	<0.001*
TMT-B§	No-Diabetes (n=4080)	Ref.		Ref.	
	Diabetes (n=1749)	0.17 (0.11, 0.22)	<0.001*	0.12 (0.07, 0.17)	<0.001*

GCF, Global Cognitive Function; MMSE, Mini-Mental State Examination; CDT, Clock Drawing Test; DST-f, Digit Span Test forward section; DST-b, Digit Span Test backward section; VFT-a, Verbal Fluency Test animal category; VFT-p, Verbal Fluency Test letter “p”; TMT-A, Trail Making Test part A; TMT-B, Trail Making Test part B.

§ Inverse neuropsychological assessment score.

Model 1: adjusted for sex, age (in years), intervention group, and center size (<250; 250-300, 300-400; ≥400)

Model 2: further adjusted for baseline education level (primary school; secondary school; college), civil status (single, divorced or separated; married; widower), physical activity (MET min/week), smoking habits (smoker; former smoker; never smoker), alcohol intake (g/day, adding the quadratic term), 17-point Mediterranean diet score, BMI (kg/m^2), hypertension (yes/no), hypercholesterolemia (yes/no), and depressive symptomatology (yes/no).

Beta coefficients were estimated using linear regression models with robust standard errors to account for intracluster correlations.

* Significant association after Benjamini-Hochberg correction .

Supplementary Table 2 Longitudinal cognitive impairment incidence in participants with normal cognitive performance at baseline.

Cognitive assessments	Diabetes status	Model 1		Model 2	
		Odds ratio (95% CI)	P-value	Odds Ratio (95% CI)	P-value
GCF	No-Diabetes (n=3131)	Ref.		Ref.	
	Diabetes (n=1191)	1.43 (1.03, 1.99)	0.031	1.34 (0.96, 1.87)	0.084
MMSE	No-Diabetes (n=3801)	Ref.		Ref.	
	Diabetes (n=1613)	1.44 (0.99, 2.08)	0.054	1.38 (0.95, 2.01)	0.094
CDT	No-Diabetes (n=3558)	Ref.		Ref.	
	Diabetes (n=1510)	1.17 (0.94, 1.44)	0.153	1.11 (0.90, 1.38)	0.331
DST-f	No-Diabetes (n=3403)	Ref.		Ref.	
	Diabetes (n=1328)	0.96 (0.50, 1.87)	0.913	0.88 (0.46, 1.66)	0.688
DST-b	No-Diabetes (n=3570)	Ref.		Ref.	
	Diabetes (n=1409)	1.07 (0.52, 2.17)	0.856	1.04 (0.51, 2.15)	0.909
VFT-a	No-Diabetes (n=3921)	Ref.		Ref.	
	Diabetes (n=1677)	1.39 (1.08, 1.79)	0.011	1.30 (1.01, 1.68)	0.048
VFT-p	No-Diabetes (n=3898)	Ref.		Ref.	
	Diabetes (n=1684)	1.08 (0.80, 1.46)	0.607	1.02 (0.75, 1.39)	0.908
TMT-A§	No-Diabetes (n=3864)	Ref.		Ref.	
	Diabetes (n=1625)	1.30 (0.96, 1.75)	0.090	1.23 (0.91, 1.67)	0.177
TMT-B§	No-Diabetes (n=3676)	Ref.		Ref.	
	Diabetes (n=1492)	1.24 (0.98, 1.58)	0.076	1.19 (0.93, 1.53)	0.170

GCF, Global Cognitive Function; MMSE, Mini-Mental State Examination; CDT, Clock Drawing Test; DST-f, Digit Span Test forward section; DST-b, Digit Span Test backward section; VFT-a, Verbal Fluency Test animal category; VFT-p, Verbal Fluency Test letter “p”; TMT-A, Trail Making Test part A; TMT-B, Trail Making Test part B.

§ Inverse neuropsychological assessment score.

Cognitive impairment was defined by the dichotomization of neuropsychological assessments at respective baseline and follow-up visit (GCF, impaired ≤ 10th percentile; MMSE, impaired ≤ 24; CDT, impaired ≤ 4; VFT-a, VFT-p, DST-d, DST-b, impaired ≤ respective mean - 1.5 SD; TMT-A, TMT-B ≥ respective mean + 1.5 SD)

Model 1: adjusted for sex, age (in years), intervention group, and center size (<250; 250-300, 300-400; ≥400)

Model 2: further adjusted for baseline education level (primary school; secondary school; college), civil status (single, divorced or separated; married; widower), physical activity (MET min/week), smoking habits (smoker; former smoker; never smoker), alcohol intake (g/day, adding the quadratic term), 17-point Mediterranean diet score, BMI (kg/m²), hypertension (yes/no), hypercholesterolemia (yes/no), and depressive symptomatology (yes/no).

Odd ratios were estimated using logistic regression models with robust standard errors to account for intracluster correlations.

Supplementary Table 3 Association between baseline diabetes control and changes in cognitive Z-scores.

Z-scores	Diabetes control	Model 1		Model 2	
		β (95% CI)	P-value	β (95% CI)	P-value
GCF	Good (n=761)	Ref.		Ref.	
	Poor (n=590)	-0.14 (-0.23, -0.04)	0.006*	-0.07 (-0.16, 0.01)	0.089
MMSE	Good (n=957)	Ref.		Ref.	
	Poor (n=755)	-0.08 (-0.17, 0.01)	0.091	-0.04 (-0.14, 0.05)	0.338
CDT	Good (n=958)	Ref.		Ref.	
	Poor (n=754)	-0.02 (-0.11, 0.07)	0.672	0.01 (-0.09, 0.10)	0.940
DST-f	Good (n=799)	Ref.		Ref.	
	Poor (n=619)	-0.05 (-0.15, 0.05)	0.341	-0.02 (-0.12, 0.07)	0.674
DST-b	Good (n=799)	Ref.		Ref.	
	Poor (n=619)	-0.04 (-0.13, 0.06)	0.480	-0.01 (-0.10, 0.09)	0.939
VFT-a	Good (n=983)	Ref.		Ref.	
	Poor (n=776)	-0.11 (-0.20, -0.02)	0.013	-0.07 (-0.16, 0.01)	0.090
VFT-p	Good (n=983)	Ref.		Ref.	
	Poor (n=776)	-0.18 (-0.27, -0.09)	<0.001*	-0.13 (-0.22, -0.04)	0.003*
TMT-A§	Good (n=982)	Ref.		Ref.	
	Poor (n=773)	0.10 (0.01, 0.20)	0.031	0.06 (-0.03, 0.15)	0.212
TMT-B§	Good (n=981)	Ref.		Ref.	
	Poor (n=768)	0.07 (-0.03, 0.17)	0.136	0.01 (-0.08, 0.10)	0.820

GCF, Global Cognitive Function; MMSE, Mini-Mental State Examination; CDT, Clock Drawing Test; DST-f, Digit Span Test forward section; DST-b, Digit Span Test backward section; VFT-a, Verbal Fluency Test animal category; VFT-p, Verbal Fluency Test letter “p”; TMT-A, Trail Making Test part A; TMT-B, Trail Making Test part B.

§ Inverse neuropsychological assessment score.

Diabetes control was assessed in participants with diabetes.

Good control when HbA1c was ≤57 mmol/mol (≤7%); Poor control when HbA1c was ≥57 mmol/mol (≥7%)

Model 1: adjusted for sex, age (in years), intervention group, and center size (<250; 250-300, 300-400; ≥400)

Model 2: further adjusted for baseline education level (primary school; secondary school; college), civil status (single, divorced or separated; married; widower), physical activity (MET min/week), smoking habits (smoker; former smoker; never smoker), alcohol intake (g/day, adding the quadratic term), 17-point Mediterranean diet score, BMI (kg/m^2), hypertension (yes/no), hypercholesterolemia (yes/no), and depressive symptomatology (yes/no).

Beta coefficients were estimated using linear regression models with robust standard errors to account for intracluster correlations.

* Significant association after Benjamini-Hochberg correction.

Supplementary Table 4 Association between baseline insulin treatment and changes in cognitive Z-scores.

Z-scores	Insulin treatment	Model 1		Model 2		Model 3	
		β (95% CI)	P-value	β (95% CI)	P-value	β (95% CI)	P-value
GCF	No (n=1153)	Ref.		Ref.		Ref.	
	Yes (n=198)	-0.33 (-0.48, -0.18)	<0.001*	-0.31 (-0.44, -0.18)	<0.001*	-0.28 (-0.42, -0.15)	<0.001*
MMSE	No (n=1456)	Ref.		Ref.		Ref.	
	Yes (n=256)	-0.05 (-0.18, 0.09)	0.508	-0.01 (-0.14, 0.13)	0.940	0.01 (-0.13, 0.15)	0.940
CDT	No (n=1456)	Ref.		Ref.		Ref.	
	Yes (n=256)	-0.11 (-0.24, 0.02)	0.102	-0.09 (-0.22, 0.04)	0.177	-0.07 (-0.21, 0.07)	0.299
DST-f	No (n=1211)	Ref.		Ref.		Ref.	
	Yes (n=207)	-0.23 (-0.36, -0.09)	0.001*	-0.23 (-0.35, -0.10)	<0.001*	-0.24 (-0.38, -0.11)	<0.001*
DST-b	No (n=1211)	Ref.		Ref.		Ref.	
	Yes (n=207)	-0.18 (-0.31, -0.04)	0.010*	-0.16 (-0.28, -0.04)	0.011*	-0.17 (-0.30, -0.04)	0.011*
VFT-a	No (n=1495)	Ref.		Ref.		Ref.	
	Yes (n=264)	-0.18 (-0.31, 0.05)	0.005*	-0.15 (-0.27, -0.03)	0.013*	-0.12 (-0.24, 0.01)	0.066
VFT-p	No (n=1495)	Ref.		Ref.		Ref.	
	Yes (n=264)	-0.22 (-0.34, -0.09)	0.001*	-0.19 (-0.31, -0.07)	0.002*	-0.15 (-0.27, -0.02)	0.024*
TMT-A§	No (n=1492)	Ref.		Ref.		Ref.	
	Yes (n=263)	0.25 (0.10, 0.39)	0.001*	0.20 (0.06, 0.34)	0.006*	0.17 (0.02, 0.33)	0.031*
TMT-B§	No (n=1488)	Ref.		Ref.		Ref.	
	Yes (n=261)	0.25 (0.11, 0.39)	<0.001*	0.20 (0.07, 0.33)	0.003*	0.19 (0.05, 0.33)	0.008*

GCF, Global Cognitive Function; MMSE, Mini-Mental State Examination; CDT, Clock Drawing Test; DST-f, Digit Span Test forward section; DST-b, Digit Span Test backward section; VFT-a, Verbal Fluency Test animal category; VFT-p, Verbal Fluency Test letter “p”; TMT-A, Trail Making Test part A; TMT-B, Trail Making Test part B.

§ Inverse neuropsychological assessment score.

Insulin treatment was assessed in participants with diabetes.

Model 1: adjusted for sex, age (in years), intervention group, and center size (<250; 250-300, 300-400; ≥400).

Model 2: further adjusted for baseline education level (primary school; secondary school; college), civil status (single, divorced or separated; married; widower), physical activity (MET min/week), smoking habits (smoker; former smoker; never smoker), alcohol intake (g/day, adding the quadratic term), 17-point Mediterranean diet score, BMI (kg/m²), hypertension (yes/no), hypercholesterolemia (yes/no), and depressive symptomatology (yes/no).

Model 3: further adjusted for baseline diabetes control (good; poor); diabetes duration (<5-year diabetes duration; ≥5-year diabetes duration).

Beta coefficients were estimated using linear regression models with robust standard errors to account for intracluster correlations.

* Significant association after Benjamini-Hochberg correction.

Supplementary Table 5 Association between baseline sulfonylureas treatment and changes in cognitive Z-scores.

Z-scores	Sulfonylureas treatment	Model 1		Model 2		Model 3	
		β (95% CI)	P-value	β (95% CI)	P-value	β (95% CI)	P-value
GCF	No (n=1184)	Ref.		Ref.		Ref.	
	Yes (n=167)	-0.13 (-0.28, 0.02)	0.087	-0.07 (-0.20, 0.06)	0.269	-0.03 (-0.17, 0.10)	0.650
MMSE	No (n=1494)	Ref.		Ref.		Ref.	
	Yes (n=218)	-0.05 (-0.19, 0.09)	0.479	-0.03 (-0.17, 0.10)	0.631	-0.02 (-0.17, 0.12)	0.735
CDT	No (n=1 493)	Ref.		Ref.		Ref.	
	Yes (n=219)	-0.09 (-0.23, 0.04)	0.183	-0.08 (-0.22, 0.06)	0.240	-0.07 (-0.21, 0.08)	0.350
DST-f	No (n=1243)	Ref.		Ref.		Ref.	
	Yes (n=175)	-0.06 (-0.21, 0.09)	0.415	-0.02 (-0.16, 0.13)	0.817	-0.01 (-0.16, 0.14)	0.893
DST-b	No (n=1243)	Ref.		Ref.		Ref.	
	Yes (n=175)	-0.01 (-0.15, 0.14)	0.917	0.05 (-0.09, 0.19)	0.504	0.06 (-0.09, 0.20)	0.441
VFT-a	No (n=1534)	Ref.		Ref.		Ref.	
	Yes (n=225)	-0.03 (-0.16, 0.10)	0.649	0.01 (-0.12, 0.14)	0.893	0.05 (-0.08, 0.18)	0.480
VFT-p	No (n=1534)	Ref.		Ref.		Ref.	
	Yes (n=225)	-0.14 (-0.28, -0.01)	0.047	-0.10 (-0.22, 0.03)	0.134	-0.05 (-0.18, 0.07)	0.406
TMT-A§	No (n=1530)	Ref.		Ref.		Ref.	
	Yes (n=225)	0.26 (0.09, 0.43)	0.003	0.22 (0.07, 0.38)	0.005	0.20 (0.04, 0.36)	0.015
TMT-B§	No (n=1525)	Ref.		Ref.		Ref.	
	Yes (n=224)	0.14 (-0.01, 0.28)	0.054	0.09 (-0.03, 0.22)	0.156	0.09 (-0.06, 0.20)	0.280

GCF, Global Cognitive Function; MMSE, Mini-Mental State Examination; CDT, Clock Drawing Test; DST-f, Digit Span Test forward section; DST-b, Digit Span Test backward section; VFT-a, Verbal Fluency Test animal category; VFT-p, Verbal Fluency Test letter “p”; TMT-A, Trail Making Test part A; TMT-B, Trail Making Test part B

§ Inverse neuropsychological assessment score.

Sulfonylureas treatment was assessed in participants with diabetes.

Model 1: adjusted for sex, age (in years), intervention group, and center size (<250; 250-300, 300-400; ≥400).

Model 2: further adjusted for baseline education level (primary school; secondary school; college), civil status (single, divorced or separated; married; widower), physical activity (MET min/week), smoking habits (smoker; former smoker; never smoker), alcohol intake (g/day, adding the quadratic term), 17-point Mediterranean diet score, BMI (kg/m²), hypertension (yes/no), hypercholesterolemia (yes/no), and depressive symptomatology (yes/no).

Model 3: further adjusted for baseline diabetes control (good; poor); diabetes duration (<5-year diabetes duration; ≥5-year diabetes duration).

Beta coefficients were estimated using linear regression models with robust standard errors to account for intracluster correlations.

Supplementary Table 6 Association between baseline metformin treatment and changes in cognitive Z-scores.

Z-scores	Metformin treatment	Model 1		Model 2		Model 3	
		β (95% CI)	P-value	β (95% CI)	P-value	β (95% CI)	P-value
GCF	No (n=313)	Ref.		Ref.		Ref.	
	Yes (n=1038)	0.03 (-0.09, 0.15)	0.615	0.04 (-0.06, 0.15)	0.441	0.07 (-0.04, 0.18)	0.202
MMSE	No (n=400)	Ref.		Ref.		Ref.	
	Yes (n=1312)	-0.02 (-0.13, 0.09)	0.701	-0.02 (-0.13, 0.09)	0.718	-0.02 (-0.13, 0.09)	0.764
CDT	No (n=398)	Ref.		Ref.		Ref.	
	Yes (n=1314)	0.04 (-0.07, 0.15)	0.466	0.05 (-0.06, 0.16)	0.374	0.07 (-0.05, 0.18)	0.245
DST-f	No (n=322)	Ref.		Ref.		Ref.	
	Yes (n=1096)	-0.03 (-0.14, 0.09)	0.674	-0.01 (-0.12, 0.11)	0.902	-0.01 (-0.12, 0.11)	0.950
DST-b	No (n=322)	Ref.		Ref.		Ref.	
	Yes (n=1096)	0.05 (-0.07, 0.16)	0.425	0.07 (-0.04, 0.18)	0.185	0.08 (-0.03, 0.19)	0.159
VFT-a	No (n=403)	Ref.		Ref.		Ref.	
	Yes (n=1356)	-0.07 (-0.17, 0.04)	0.208	-0.06 (-0.15, 0.04)	0.261	-0.04 (-0.14, 0.06)	0.453
VFT-p	No (n=403)	Ref.		Ref.		Ref.	
	Yes (n=1356)	-0.04 (-0.15 0.07)	0.517	-0.03 (-0.13, 0.07)	0.557	-0.01 (-0.11, 0.09)	0.862
TMT-A§	No (n=403)	Ref.		Ref.		Ref.	
	Yes (n=1352)	0.01 (-0.11, 0.12)	0.919	0.01 (-0.11, 0.12)	0.924	-0.02 (-0.14, 0.10)	0.785
TMT-B§	No (n=401)	Ref.		Ref.		Ref.	
	Yes (n=1348)	-0.01 (-0.12, 0.11)	0.904	-0.01 (-0.12, 0.10)	0.845	-0.03 (-0.14, 0.08)	0.587

GCF, Global Cognitive Function; MMSE, Mini-Mental State Examination; CDT, Clock Drawing Test; DST-f, Digit Span Test forward section; DST-b, Digit Span Test backward section; VFT-a, Verbal Fluency Test animal category; VFT-p, Verbal Fluency Test letter “p”; TMT-A, Trail Making Test part A; TMT-B, Trail Making Test part B

§ Inverse neuropsychological assessment score.

Metformin treatment was assessed in participants with diabetes.

Model 1: adjusted for sex, age (in years), intervention group, and center size (<250; 250-300, 300-400; ≥400).

Model 2: further adjusted for baseline education level (primary school; secondary school; college), civil status (single, divorced or separated; married; widower), physical activity (MET min/week), smoking habits (smoker; former smoker; never smoker), alcohol intake (g/day, adding the quadratic term), 17-point Mediterranean diet score, BMI (kg/m²), hypertension (yes/no), hypercholesterolemia (yes/no), and depressive symptomatology (yes/no).

Model 3: further adjusted for baseline diabetes control (good; poor); diabetes duration (<5-year diabetes duration; ≥5-year diabetes duration).

Beta coefficients were estimated using linear regression models with robust standard errors to account for intracluster correlations.

Supplementary Table 7 Association between baseline IDPP-4 treatment and changes in cognitive Z-scores.

Z-scores	IDPP4 treatment	Model 1		Model 2		Model 3	
		β (95% CI)	P-value	β (95% CI)	P-value	β (95% CI)	P-value
GCF	No (n=1010)	Ref.		Ref.		Ref.	
	Yes (n=341)	-0.05 (-0.16, 0.07)	0.434	-0.03 (-0.13, 0.07)	0.565	0.01 (-0.10, 0.12)	0.827
MMSE	No (n=1296)	Ref.		Ref.		Ref.	
	Yes (n=416)	0.01 (-0.11, 0.11)	0.961	0.01 (-0.09, 0.12)	0.814	0.02 (-0.09, 0.13)	0.704
CDT	No (n=1297)	Ref.		Ref.		Ref.	
	Yes (n=415)	-0.05 (-0.17, 0.06)	0.361	-0.05 (-0.17, 0.06)	0.353	-0.04 (-0.16, 0.08)	0.551
DST-f	No (n=1062)	Ref.		Ref.		Ref.	
	Yes (n=356)	0.06 (-0.06, 0.18)	0.327	0.06 (-0.05, 0.18)	0.280	0.08 (-0.04, 0.20)	0.214
DST-b	No (n=1062)	Ref.		Ref.		Ref.	
	Yes (n=356)	0.07 (-0.05, 0.19)	0.266	0.08 (-0.04, 0.19)	0.193	0.09 (-0.03, 0.20)	0.130
VFT-a	No (n=1336)	Ref.		Ref.		Ref.	
	Yes (n=423)	-0.05 (-0.15, 0.05)	0.341	-0.04 (-0.14, 0.05)	0.391	-0.01 (-0.11, 0.09)	0.792
VFT-p	No (n=1336)	Ref.		Ref.		Ref.	
	Yes (n=423)	-0.05 (-0.16, 0.06)	0.365	-0.04 (-0.15, 0.06)	0.423	-0.01 (-0.11, 0.10)	0.910
TMT-A§	No (n=1333)	Ref.		Ref.		Ref.	
	Yes (n=422)	0.05 (-0.05, 0.16)	0.341	0.04 (-0.06, 0.14)	0.459	0.01 (-0.10, 0.11)	0.889
TMT-B§	No (n=1327)	Ref.		Ref.		Ref.	
	Yes (n=422)	0.02 (-0.09, 0.13)	0.702	0.01 (-0.10, 0.11)	0.928	-0.02 (-0.13, 0.08)	0.692

IDPP-4, dipeptidyl peptidase-4 inhibitors; GCF, Global Cognitive Function; MMSE, Mini-Mental State Examination; CDT, Clock Drawing Test; DST-f, Digit Span Test forward section; DST-b, Digit Span Test backward section; VFT-a, Verbal Fluency Test animal category; VFT-p, Verbal Fluency Test letter “p”; TMT-A, Trail Making Test part A; TMT-B, Trail Making Test part B.

§ Inverse neuropsychological assessment score.

IDDP-4 treatment was assessed in participants with diabetes.

Model 1: adjusted for sex, age (in years), intervention group, and center size (<250; 250-300, 300-400; ≥400).

Model 2: further adjusted for baseline education level (primary school; secondary school; college), civil status (single, divorced or separated; married; widower), physical activity (MET min/week), smoking habits (smoker; former smoker; never smoker), alcohol intake (g/day, adding the quadratic term), 17-point Mediterranean diet score, BMI (kg/m²), hypertension (yes/no), hypercholesterolemia (yes/no), and depressive symptomatology (yes/no).

Model 3: further adjusted for baseline diabetes control (good; poor); diabetes duration (<5-year diabetes duration; ≥5-year diabetes duration).

Beta coefficients were estimated using linear regression models with robust standard errors to account for intracluster correlations.

APPENDIX

List of PREDIMED-Plus study investigators

Steering Committee:

J. Salas-Salvadó (Coordinator), M.A. Martínez-González, M. Fitó. E. Ros, FJ. Tinahones, D. Corella and R. Estruch.

Executive Committee:

J. Salas-Salvadó, M.A. Martínez-González, D. Corella, M. Fitó, J. Vioque, D. Romaguera, J.A Martínez, J. Wärnberg, J. Lopez-Miranda, R. Estruch, A. Bueno-Cavanillas, Á.M. Alonso-Gómez, J.A. Tur, FJ. Tinahones, L. Serra-Majem, V. Martin, J. Lapetra, C. Vázquez, X. Pinto, J. Vidal, L. Daimiel, M. Delgado-Rodríguez, M.A. Rubio and E. Ros.

Dietary and Lifestyle Intervention Committee:

J. Salas-Salvadó (chair), M.A. Martínez-González, M. Fitó and R. Estruch;

Dietary Intervention: J. Salas-Salvadó (chair), N. Babio, E. Ros, A. Sánchez-Tainta;

Physical Exercise: M. Fitó (chair), H. Schröder, A. Marcos, D. Corella, J. Warnberg;

Behavioural support: R. Estruch (chair), F. Fernández-Aranda, C. Botella and J. Salas-Salvadó.

Clinical Event Ascertainment Committee:

F. Arós (Chair), M. Aldamiz, A. Alonso-Gómez, L. Forga, A. García-Layana, J. Portu, J. Timiraos, A. González-Pinto, I. Zorrilla, M. Martínez-Kareaga, P. Seoane.

Chair: Dr. Fernando Arós

Cardiology: Dr. Angel Alonso-Gómez; Dr. Fernando Arós

Neurology: Dr. Juan Timiraos

Internal Medicine: Dr. Mikel Aldamiz; Dr. Joseba Portu

Endocrinology: Dr. Lluis Forga

Ophthalmology: Dr. Alfredo García-Layana

Psychiatry: Dr. Ana González Pinto; Dr. Iñaki Zorrilla

Oncology: Dr. Mireia Martínez; Dr. Patricia Seoane

Rovira i Virgili University, Department of Biochemistry and Biotechnology, Human Nutrition Unit, University Hospital of Sant Joan de Reus, Pere Virgili Institute for Health Research, Reus, Spain: R. Pedret Llaberia, R. Gonzalez, R. Sagarra Álamo, F. París Palleja, J. Balsells, J.M. Roca, T. Basora Gallisa, J. Vizcaino, P. Llobet Alpizarte, C. Anguera Perpiñá, M. Llauradó Vernet, C. Caballero, M. Garcia Barco, M.D. Morán Martínez, J. García Rosselló, A. Del Pozo, C. Poblet Calaf, P. Arcelin Zabal, X. Floresví, M. Ciutat Benet, A. Palau Galindo, J.J. Cabré Vila, F. Dolz Andrés, M. Soler, M. Gracia Vidal, J. Vilalta J. Boj Casajuana, M. Ricard, F. Saiz, A. Isach, M. Sanchez Marin Martinez, E. Granado Font, C, Lucena Luque, C. Mestres Sola, N. Babio, N. Becerra-Tomás, J. Basora, G. Rosique-Esteban, S. Chig, I. Abellán Cano, V. Ruiz García, C. Gomez-Martinez, L. Lopez-Gonzalez, A. Salas-Huetos, I. Paz-Graniel, J. Roig Vallverdú, C. Miñana Garcia, L. Sánchez Niembro, S. Manzanedo, J. Muralidharan, A. Atzeni, C. Valle. M, Fernández de la Puente, T. Garcidueñas-Fimbres, J. Ni, N. Khoury.

Department of Preventive Medicine and Public Health, University of Navarra-Navarra Institute for Health Research (IdiSNA), Pamplona, Spain: M. Ruiz-Canela, E. Toledo, P. Buil-Cosiales, Z. Vázquez, C. Razquin, M. Bes-Rastrollo, A. Gea, A. Sanchez Tainta, B. SanJulian Aranguren, E. Goñi, L. Goñi, M.J. Cobo, A. Rico-Campa, F.J. Basterra Gortari, A. Garcia Arellano, J. Diez-Espino, O. Lecea-Juarez, J. Carlos Cenoz-Osinaga, I. Alvarez-Alvarez, M.C. Sayon-Orea, C.I. Fernandez-Lázaro, L. Ruiz-Estigarribia, J. Bartolome-Resano, A. Sola-Larraza (†), E. Lozano-Oloriz, B. Cano-Valles, S. Eguaras, E. Pascual Roquet-Jalmar, I. Galilea-Zabalza, H. Lancova, R. Ramallal, M.L. Garcia-Perez, V. Estremera-Urabayen, M.J. Ariz-Arnedo, C. Hijos-Larraz, C. Fernandez-Alfaro, B. Iñigo-Martinez, R. Villanueva Moreno, S. Martin-Almendros, L. Barandiaran-Bengoetxea, C. Fuertes-Goñi, A. Lezaun-Indurain, M.J. Guruchaga-Arcelus, O. Olmedo-Cruz, L. Escriche-Erviti, R. Ansorena-Ros, R. Sanmatin-Zabaleta, J. Apalategi-Lasa, J. Villanueva-Telleria, M.M. Hernández-Espinosa, L. Herrera-Valdez, L. Dorronsoro-Dorronsoro, Lourdes Echeverria-Lizarraga (†), J.A. Cabeza-Beunza, P. Fernández-Urretavizcaya, P. Gascó-García, C. Royo-Jimenez, J. Moran-Pí, F. Salazar-Fernández, F.J. Chasco-Ros, F. Cortés-Ugalde, J.J. Jurio-Burgui, P. Pascual-Pascual, A.I. Rodríguez-Ezpeleta, M. Esparza-Cáceres, C. Arroyo-Azpa, M. Rodríguez-Sanz de Galdeano, T. Forcen-Alonso, M. Armendariz-Marcotegui, A. Brugos-Larumbe, A. Arillo, B. López-Aisa.

Department of Preventive Medicine, University of Valencia, University Jaume I, Conselleria de Sanitat de la Generalitat Valenciana, Valencia, Spain: J.I. González, J.V. Sorlí, O. Portolés, R. Fernández-Carrión, C. Ortega-Azorín, R. Barragán, E.M. Asensio, O. Coltell, R. Martínez-Lacruz, I. Giménez-Alba, C. Sáiz, R. Osma, E. Férriz, I. González-Monje, P. Guillém-Sáiz, F. Giménez-Fernández, L. Quiles, P. Carrasco, A. Carratalá-Calvo, C. Valero-Barceló, C. Mir, S. Sánchez-Navarro, J. Navas, I. González-Gallego, L. Bort-Llorca, L. Pérez-Ollero, M. Giner-Valero, R. Monfort-Sáez, J. Nadal-Sayol, V. Pascual-Fuster, M. Martínez-Pérez, C. Riera, M.V. Belda, A. Medina, E. Miralles, M.J. Ramírez-Esplugues, M. Rojo-Furió, G. Mattingley, M.A. Delgado, M.A. Pages, Y Riofrío, L. Abuomar, N. Blasco-Lafarga, R. Tosca, L. Lizán, A.M Valcarce, M.D. Medina, S. de Valcárcel, N. Tormo, O. Felipe-Román, S. Lafuente, E.I. Navío, G. Aldana, J.V. Crespo, J.L. Llosa, L. González-García, R. Raga-Marí.

Cardiovascular Risk and Nutrition Research Group, Endocrinology Service, Neurosciences Programme, Clinical Research Unit at the Hospital del Mar Medical Research Institute (IMIM), Barcelona. Medicine Departament, Universitat Autònoma de Barcelona, Barcelona, Spain: M. Fitó, O. Castañer, M.A. Muñoz, M.D. Zomeño, A. Hernaéz, L. Torres, M. Quifer, R. Llimona, G Freixer, KA. Pérez-Vega, M. Farràs, R. Elosua, J. Vila, I. Subirana, S. Pérez, A. Goday, J.J. Chillaron Jordan, J.A. Flores Lerroux, D. Benaiges Boix, G. Llauradó, M. Farré, E. Menoyo, A. Aldea-Perona, M. Pérez-Otero, D. Muñoz-Aguayo, S. Gaixas, G. Blanchart, A. Sanllorente, M. Soria, J. Valussi, A. Cuenca, L. Forcano, A. Pastor, A. Boronat, S. Tello, M. Cabañero, L. Franco, H. Schröder, R. De la Torre, C. Medrano, J. Bayó, M.T. García, V. Robledo, P. Babi, E. Canals, N. Soldevila, L. Carrés, C. Roca, M.S. Comas, G. Gasulla, X. Herraiz, A. Martínez, E. Vinyoles, J.M. Verdú, M. Masague Aguade, E. Baltasar Massip, M. López Grau, M. Mengual, V. Moldon, M. Vila Vergaz, R. Cabanes Gómez, Ciurana, M. Gili Riu, A. Palomeras Vidal, F Peñas F, A Raya, M.A. Sebastian, M. Valls, J. Guerrero, M. Marne, E. Minguella, M. Montenegro, A. Sala, M.R. Senan, N. Talens, N. Vera.

Nutritional Epidemiology Unit, Miguel Hernandez University, ISABIAL-FISABIO, Alicante, Spain: J. Vioque, M. García-de-la-Hera, S. Gonzalez-Palacios, L. Torres-Collado, L. Compañ-Gabucio, A. Oncina-Canovas, L. Notario-Barandiaran, D. Orozco-Beltran, S. Pertusa Martínez, A. Asencio, I. Candela-García, J.M. Zazo, D. Vivancos Aparicio, N. Fernández-Brufal, J. Román Maciá, F. Ortiz Díaz, M. García Muñoz, C. Barceló, E. Martínez-García, M Damaj-Hamieh, M.C. Martínez Vergara, M.A. Sempere Pascual, S.J. Miralles Gisbert, A. González Botella, C.M. López García, R. Valls Enguix, N. Gómez Bellvert, V. Martínez Avilés, R. Lloret Macián, A. Pastor Morel, M. Mayor-

Llorca, J.J. Ballester Baixauli, G. Notario García, M.A. Belmar-Bueno, E.P. Cases Pérez, C. Tercero Maciá, L.A. Mira Castejón, J. Torregrosa García, C. Pastor Polo, E. Puig Agulló, M.V. Hernándis Marsán, M.J. González Fajardo, I. Hervella Durantez, M.C. Latorre Use, A. Bernabé Casanova, F. Medina Ruzafa, E. Robledano, I. Vilanova Martínez, A. Molina Santiago.

Hospital Son Espases (HUSE) and Institute for Health Research Illes Balears (IdISBa), Palma de Mallorca, Spain: M. Fiol, M. Moñino, A. Colom, J. Konieczna, M. Morey, A.M. Galmés-Panadés, M.A. Martín, E. Rayó, J. Llobera, J. Fernández-Palomeque, E. Fortuny, M. Noris, L. López, X. Rosselló, S. Munuera, F. Tomás, F. Fiol, A. Jover, J.M. Janer, C. Vallespir, I. Mattei, N. Feuerbach, M. del Mar Sureda, S. Vega, L. Quintana, A. Fiol, M. Amador, S. González, J. Coll, A. Moyá, T. Piqué Sistac, M.D. Sanmartín Fernández, M.C. Piña Valls, M.A. Llorente San Martín, J. Pou Bordoy.

Department of Nutrition, Food Sciences, and Physiology, Center for Nutrition Research, University of Navarra, Pamplona, Spain: I. Abete, I. Cantero, C. Cristobo, I. Ibero-Baraibar, M. Zulet, J. Ágreda-Peiró, M.D. Lezáun-Burgui, N. Goñi-Ruiz, R. Bartolomé-Resano, E. Cano-Cáceres, T. Elcarte-López, E. Echarte-Osacain, B. Pérez-Sanz, I. Blanco-Platero, A. Andueza- Azcárate, A. Gimeno-Aznar, E. Ursúa-Sesma, B. Ojeda-Bilbao, J. Martinez-Jarauta, L. Ugalde-Sarasa, B. Rípodas-Echarte, M.V. Güeto-Rubio, C. Napal-Lecumberri, MD Martínez-Mazo, E Arina-Vergara, A. Parra-Osés, F. Artal-Moneva, F. Bárcena-Amigo, F. Calle-Irastoza, J. Abad-Vicente, J.I. Armendáriz-Artola, P. Iñigo-Cibrian, J. Escribano-Jarauta, J. Ulibarri-delportillo, B. Churio-Beraza, Y. Monzón-Martínez, E. Madoz-Zubillaga, C. Arroniz.

University of Málaga and Institute of Biomedical Research in Malaga (IBIMA), Málaga, Spain: F.J. Barón-López, J.C. Fernández García, N. Pérez-Farinós, N. Moreno-Morales, M. del C. Rodríguez-Martínez, J. Pérez-López, J.C. Benavente-Marín, E. Crespo Oliva, E. Contreras Fernández, F.J. Carmona González, R. Carabaño Moral, S. Torres Moreno, M.V. Martín Ruíz, M. Alcalá Cornide, V. Fuentes Gómez.

Lipids and Atherosclerosis Unit, Department of Internal Medicine, Maimonides Biomedical Research Institute of Cordoba (IMIBIC), Reina Sofia University Hospital, University of Cordoba, Cordoba, Spain: J. López-Miranda, A. Garcia-Rios, J. Criado García, A.I. Jiménez Morales, A. Ortiz Morales, J.D. Torres Peña, F.J. Gómez Delgado, J.F. Alcalá, A. León Acuña, A.P. Arenas Larriva, F. Rodríguez Cantalejo, J. Caballero Villaraso, I. Nieto Eugenio, P. Coronado Carvajal, M.C del Campo Molina, P.J. Peña Orihuela, I. Perez Corral, G. Quintana Navarro.

Department of Internal Medicine, Institut d'Investigacions Biomèdiques August Pi i Sunyer (IDIBAPS), Hospital Clínic, University of Barcelona, Barcelona, Spain: R. Casas, M. Domenech, C. Viñas, S. Castro-Barquero, A.M. Ruiz-León, R. Losno, L. Tarés, A. Jordán, R. Soriano, M. Camafort, C. Sierra, E. Sacanella, A. Sala-Vila, J. M. Cots, I. Sarroca, M. García, N. Bermúdez, A. Pérez, I. Duaso, A. de la Arada, R. Hernández, C. Simón, M.A. de la Poza, I. Gil, M. Vila, C. Iglesias, N. Assens, M. Amatller, LL. Rams, T. Benet, G. Fernández, J. Teruel, A. Azorin, M. Cubells, D. López, J.M. Llovet, M.L. Gómez, P. Climente, L. de Paula, J. Soto, C. Carbonell, C. Llor, X. Abat, A. Cama, M. Fortuny, C. Domingo, A. I. Liberal, T. Martínez, E. Yañez, M. J. Nieto, A. Pérez, E. Lloret, C. Carrazoni, A. M. Belles, C. Olmos, M. Ramentol, M. J. Capell, R. Casas, I. Giner, A. Muñoz, R. Martín, E. Moron, A. Bonillo, G. Sánchez, C. Calbó, J. Pous, M. Massip, Y. García, M.C. Massagué, R. Ibañez, J. Llaona, T. Vidal, N. Vizcay, E. Segura, C. Galindo, M. Moreno, M. Caubet, J. Altirriba, G. Fluxà, P. Toribio, E. Torrent, J. J. Anton, A. Viaplana, G. Vieytes, N. Duch, A. Pereira, M. A. Moreno, A. Pérez, E. Sant, J. Gené, H. Calvillo, F. Pont, M. Puig, M. Casasayas, A. Garrich, E. Senar, A. Martínez, I. Boix, E. Sequeira, V. Aragunde, S. Riera, M. Salgado, M. Fuentes, E. Martín, A. Ubieto, F. Pallarés, C. Sala, A. Abilla, S. Moreno, E. Mayor, T. Colom, A. Gaspar, A. Gómez, L. Palacios, R. Garrigosa.

Departament of Preventive Medicine and Public Health, University of Granada, Granada, Spain: L. García Molina, B. Riquelme Gallego, N. Cano Ibañez, A. Maldonado Calvo, A. López Maldonado, E.M. Garrido, A. Baena Dominguez, F. García Jiménez, E. Thomas Carazo, A. Jesús Turnes González, F. González Jiménez, F. Padilla Ruiz, J. Machado Santiago, M.D. Martínez Bellón, A. Pueyos Sánchez, L. Arribas Mir, R. Rodríguez Tapioles, F. Dorador Atienza, L. Baena Camus, C. Osorio Martos, D. Rueda Lozano, M. López Alcázar, F. Ramos Díaz, M. Cruz Rosales Sierra, P. Alguacil Cubero, A. López Rodriguez, F. Guerrero García, J. Tormo Molina, F. Ruiz Rodríguez.

Bioaraba Health Research Institute, Cardiovascular, Respiratory and Metabolic Area; Osakidetza Basque Health Service, Araba University Hospital; University of the Basque Country UPV/EHU, Vitoria-Gasteiz, Spain: I. Salaverria, A. Alonso-Gómez, M.C. Belló, L. Tojal, L. Goicolea, C. Sorto, A Goikoetxea, A. Casi Casanellas, M.L. Arnal Otero, J. Ortueta Martínez De Arbulo, J. Vinagre Morgado, J. Romeo Ollora, J. Urraca, M.I. Sarriegui Carrera, F.J. Toribio, E. Magán, A. Rodríguez, S. Castro Madrid, M.T. Gómez Merino, M. Rodríguez Jiménez, M. Gutiérrez Jodra, B. López Alonso, J. Iturralde Iriso, C. Pascual Romero, A. Izquierdo De La Guerra.

Research Group on Community Nutrition & Oxidative Stress, University of Balearic Islands, Palma de Mallorca, Spain: M. Abbate, E. Angullo, E. Argelich, M.M. Bibiloni, C. Bouzas, X. Capó, S. Carreres, L. Gallardo, J.M. Gámez, B. García, C. García, A. Julibert, C. Gómez, I. Llompart, A. Martorell, C.M. Mascaró, D. Mateos, M, Monserrat, S. Montemayor, A. Pons, A. Pouso, J. Ramos, V. Ramos, T. Ripoll, T. Rodríguez, L. Sanz, A. Sureda, S. Tejada, L. Ugarriza.

Virgen de la Victoria Hospital, University of Málaga, Málaga, Spain: M.R. Bernal López, M. Macías González, J. Ruiz Nava, J.C. Fernández García, A. Muñoz Garach, A. Vilches Pérez, A. González Banderas, A.V. Alarcón-Martín, M. García Ruiz de Mier, J. Alcaide Torres, A. Vargas Candela, M. León Fernández, R. Hernández Robles, S. Santamaría Fernández, J.M. Marín.

University of Las Palmas de Gran Canaria, Las Palmas, Spain: J. Álvarez-Pérez, E.M. Díaz Benítez, F. Díaz-Collado, A. Sánchez-Villegas, J. Pérez-Cabrera, L.T. Casañas-Quintana, R.B. García-Guerra, I. Bautista-Castaño, C. Ruano-Rodríguez, F. Sarmiento de la Fe, J.A. García-Pastor, B. Macías-Gutiérrez, I. Falcón-Sanabria, C. Simón-García, A.J. Santana-Santana, J.B. Álvarez-Álvarez, B.V. Díaz-González, J.M. Castillo Anzalas, R.E. Sosa-Also, J. Medina-Ponce.

Biomedicine Institute (IBIOMED); University of León, and Primary Health Care Management of León (Sacyl), León, Spain: Biomedicine Institute (IBIOMED); University of León, and Primary Health Care Management of León (Sacyl), León, Spain: S. Abajo Olea, L. Álvarez-Álvarez, M. Rubín García, A. Torres, P. Farias, N. Cubelos, A. Adlbi Sibai, M. Ajenjo, E. Carriedo Ule, M. Escobar Fernández, J.I. Ferradal García, J.P. Fernández Vázquez, C. González Quintana, F. González Rivero, M. Lavinia Popescu, J.I. López Gil, J. López de la Iglesia, A. Marcos Delgado, C. Merino Acevedo, S. Reguero Celada, M. Rodríguez Bul, E. Fernández Mielgo.

Department of Family Medicine, Distrito Sanitario Atención Primaria Sevilla, Sevilla, Spain: J.M. Santos-Lozano, L. Miró-Moriano, C. Domínguez-Espinaco, S. Vaquero-Díaz, F.J. García-Corte, A. Santos-Calonge, C. Toro-Cortés, N. Pelegrina-López, V. Urbano-Fernández, M. Ortega-Calvo, J. Lozano-Rodríguez, I. Rivera-Benítez, M. Caballero-Valderrama, P. Iglesias-Bonilla, P. Román-Torres, Y. Corchado-Albalat, L. Mellado-Martín.

Department of Endocrinology and Nutrition, Hospital Fundación Jimenez Díaz. Instituto de Investigaciones Biomédicas IISFJD. University Autonoma, Madrid, Spain: A.I. de Cos, S. Gutierrez, S. Artola, A. Galdon, I. Gonzalo.

Lipids and Vascular Risk Unit, Internal Medicine, University Hospital of Bellvitge- IDIBELL, Hospitalet de Llobregat, Barcelona, Spain: X. Pintó, A. Galera, M. Gimenez-Gracia, E. de la Cruz,

R. Figueras, M. Poch, R. Freixedas, F. Trias, I. Sarasa, M. Fanlo-Maresma, H. Lafuente, M. Liceran, A. Rodriguez-Sanchez, C. Pallarols, E. Gómez-Sanchez, V. Esteve-Luque, J. Monedero, X. Corbella, E. Corbella.

Department of Endocrinology, IDIBAPS, Hospital Clinic, University of Barcelona, Barcelona, Spain: A. Altés, I. Vinagre, C. Mestre, J. Viaplana, M. Serra, J. Vera, T. Freitas, E. Ortega, I. Pla, R. Olbeyra.

Nutritional Control of the Epigenome Group. Precision Nutrition and Obesity Program, Institute IMDEA-Food, CEI UAM+CSIC, Madrid, Spain: J.M. Ordovás, V. Micó, L. Berninches, L. Díez, M.J. Concejo, J. Muñoz, M. Adrián, Y. de la Fuente, C. Albertos, M.L. Cornejo, C. Cuesta. A. Montero., J. Aroca

Division of Preventive Medicine, University of Jaén, Jaén, Spain: J.J. Gaforio, S. Moraleda, N. Liétor, J.I. Peis, T. Ureña, M. Rueda, M.I. Ballesta.

Department of Endocrinology and Nutrition, Hospital Clínico San Carlos, Instituto de Investigación Sanitaria del Hospital Clínico San Carlos (IdISSC), Madrid, España (Spain para internacionales: C. Moreno Lopera, C. Aragoneses Isabel, M.A. Sirur Flores, M. Ceballos de Diego, T. Bescos Cáceres, Y. Peña Cereceda, M. Martínez Abad, R. Cabrera Vélez, M. González Cerrajero, M.A. Rubio Herrera, M. Torrego Ellacuría, A. Barabash Bustelo, M. Ortiz Ramos, A. Larrad Sainz.
